# Yoga for Opioid Withdrawal and Autonomic Regulation: A Randomized Clinical Trial

**DOI:** 10.1001/jamapsychiatry.2025.3863

**Published:** 2026-01-07

**Authors:** Suddala Goutham, Hemant Bhargav, Bharath Holla, Jayant Mahadevan, Ravindra P. Nagendra, Nishitha Jasti, Venkata Lakshmi Narasimha, Urvakhsh Meherwan Mehta, Shivarama Varambally, Ganesan Venkatasubramanian, Prabhat Chand, Bangalore Nanjundiah Gangadhar, Kevin P. Hill, Matcheri Keshavan, Pratima Murthy

**Affiliations:** 1 Department of Integrative Medicine, National Institute of Mental Health and Neurosciences, Bengaluru, Karnataka, India; 2 Centre for Addiction Medicine, Department of Psychiatry, National Institute of Mental Health and Neurosciences, Bengaluru, Karnataka, India; 3 Centre for Consciousness Studies, Department of Neurophysiology, National Institute of Mental Health and Neurosciences, Bengaluru, Karnataka, India; 4 Department of Psychiatry, National Institute of Mental Health and Neurosciences, Bengaluru, Karnataka, India; 5 Division of Addiction Psychiatry, Beth Israel Deaconess Medical Center, Harvard Medical School, Boston, Massachusetts; 6 Beth Israel Deaconess Medical Center and Massachusetts Mental Health Center, Harvard Medical School, Boston, Massachusetts

## Abstract

**Importance:**

Opioid withdrawal involves sympathetic hyperactivity and reduced parasympathetic tone, which standard pharmacological treatments may not adequately address, contributing to relapse vulnerability.

**Objective:**

To evaluate yoga as adjuvant therapy to accelerate opioid withdrawal recovery and assess its impact on heart rate variability, anxiety, sleep, and pain.

**Design, Setting, and Participants:**

This 2-arm, early-stage randomized clinical trial was conducted at an addiction medicine inpatient ward in India from April 30, 2023 to March 31, 2024. The outcome assessors and data analyst were blinded to group allocation. Participants included adults aged 18 to 50 years with opioid use disorder experiencing mild to moderate withdrawal symptoms (Clinical Opiate Withdrawal Scale [COWS] scores 4-24). Exclusion criteria included severe withdrawal, neurological conditions affecting autonomic function, severe psychiatric conditions, and recent yoga training. Of 68 individuals screened, 59 were randomized (30 yoga and 29 control participants).

**Intervention:**

Participants in the yoga group received 10 supervised 45-minute sessions during 14 days alongside standard buprenorphine treatment, including relaxation practices, postures, breathing techniques, and guided relaxation. Participants in the control group received standard buprenorphine treatment only.

**Main Outcomes and Measures:**

Co–primary outcomes included time to withdrawal stabilization (COWS score <4) and heart rate variability parameters. Secondary outcomes included anxiety (Hamilton Anxiety Rating Scale), sleep latency, and pain scores. Assessments were conducted at baseline (day 1) and day 15.

**Results:**

Fifty-nine participants (59 male [100%]; mean [SD] age, 25.6 [3.9] years) completed intent-to-treat analysis. Participants in the yoga group recovered faster than those in the control group (hazard ratio [HR], 4.40; 95% CI, 2.40-8.07; *P* < .001), with a median stabilization time of 5 days (95% CI, 4-6 days) for those in the yoga group vs 9 days (95% CI, 7-13 days) for the control group. Participants in the yoga group showed superior heart rate variability improvements with large effects on low frequency (LF) power (ω^2^ = 0.16), high frequency (HF) power (ω^2^ = 0.14), and LF/HF ratio (ω^2^ = 0.12); all effects were statistically significant (*P* < .001). Mediation analysis showed that increases in parasympathetic activity accounted for 23% of the treatment effect (indirect HR, 1.38; 95% CI, 1.10-2.03). Anxiety reduction was significantly greater among those in the yoga group (ω^2^ = 0.28; *P* < .001), with moderate improvements in sleep latency (a 61-minute reduction; *P* = .008) and pain (*P* = .004).

**Conclusions and Relevance:**

In this randomized clinical trial, yoga significantly accelerated opioid withdrawal recovery and improved autonomic regulation, anxiety, sleep, and pain. These findings support integrating yoga into withdrawal protocols as a neurobiologically informed intervention addressing core regulatory processes beyond symptom management.

**Trial Registration:**

Clinical Trials Registry of India Identifier: CTRI/2023/04/051302

## Key Points

**Question:**

Can yoga as adjuvant therapy accelerate opioid withdrawal recovery and improve autonomic regulation in patients with opioid use disorder?

**Findings:**

In this randomized clinical trial of 59 male participants with opioid use disorder, those receiving yoga alongside standard buprenorphine treatment achieved withdrawal stabilization 4.4 times faster than controls (median, 5 vs 9 days) and showed significant improvements in heart rate variability, anxiety, sleep, and pain measures.

**Meaning:**

In this trial, yoga significantly enhanced opioid withdrawal recovery through measurable autonomic and clinical improvements, supporting its integration into withdrawal protocols as a neurobiologically informed intervention.

## Teaser

This randomized clinical trial examines the efficacy of yoga as adjunct therapy to standard treatment for opioid withdrawal among patients with opioid use disorder.

## Introduction

Opioid use disorder (OUD) is a significant global public health challenge. In 2022, an estimated 60 million people worldwide used opioids nonmedically, yet only 1 in 11 individuals with drug use disorders received treatment.^1^ OUD is characterized by recurrent opioid use, leading to significant physical, psychological, and social problems.^2^ Opioids, derived from the opium poppy (*Papaver somniferum*) and including natural opiates (eg, morphine and codeine) plus semisynthetic and synthetic analogues, are widely used for pain management but carry high misuse potential.^2^ Opioid withdrawal represents a critical treatment period when symptom severity, dropout rates, and relapse risk are highest. Early intervention during this vulnerable phase can significantly impact long-term retention and recovery outcomes.^3^

In India, a 2019 national survey indicated 2.1% opioid use prevalence,^4^ with considerable variation across states, including notably higher rates in northeastern states.^5^ Historically, South India reported lower opioid use compared with India overall, but in recent years, the region reported marked increases in synthetic and pharmaceutical opioid misuse, particularly tapentadol and tramadol.^6^

Chronic opioid use induces neurobiological adaptations, particularly in the noradrenergic system, leading to dependence and relapse risk from intense cravings and severe withdrawal.^7^ Withdrawal involves physical symptoms (eg, diarrhea, insomnia, fever, pain, anxiety, and depression) and autonomic signs (eg, pupil dilation, runny nose, goosebumps, anorexia, yawning, nausea, vomiting, and sweating).^8^ These symptoms result from sympathetic nervous system overactivity due to dysregulated noradrenergic outflow.

Heart rate variability (HRV) is a key physiological measure reflecting sympathovagal balance. A 2024 review by Moon et al^9^ showed notable decreases in resting HRV among individuals with substance use disorder, significantly linked to increased stress levels, cravings, and symptom severity. Emerging evidence indicates that autonomic dysregulation, indexed by reduced HRV, is associated with greater craving and relapse vulnerability in OUD.^10,11^

In cross-sectional studies, patients with OUD exhibited significantly lower resting-state high-frequency HRV compared with individuals without OUD, with reduced HRV correlating with greater opioid craving.^10^ Similarly, individuals who use heroin demonstrated decreased cardiac vagal activity relative to healthy controls, alongside differential autonomic alterations under methadone treatment between patients who relapsed and those who did not.^11^ These findings suggest HRV indices may be useful tools for monitoring early relapse indications.^9^

Although buprenorphine, clonidine, and lofexidine effectively manage withdrawal and cravings, they do not adequately address sympathovagal imbalance, particularly reduced parasympathetic activity during withdrawal.^12,13^ This persistent autonomic dysregulation contributes to ongoing stress reactivity and relapse vulnerability, representing a critical gap in standard OUD management. Behavioral interventions, including cognitive behavioral therapy and mindfulness-based approaches, improve autonomic regulation as reflected in HRV.^14^ Garland et al^14^ demonstrated that mindfulness-oriented recovery enhancement increases HRV, and importantly, these HRV improvements mediated reductions in opioid use among patients with chronic pain. Similarly, cognitive behavioral therapy has been shown to increase HRV and reduce heart rate in patients with depression and coronary heart disease.^15^ However, cognitive behavioral therapy can be difficult to initiate during acute withdrawal due to high distress and impaired concentration, highlighting the need for simpler, more accessible strategies.

Yoga, encompassing physical postures, breathing techniques, meditation, and relaxation, is uniquely positioned to address the need for accessible interventions that can be initiated even during acute withdrawal by directly enhancing parasympathetic tone and promoting physiological self-regulation.^16^ This therapeutic gap provides the rationale to investigate yoga as adjunct therapy to restore sympathovagal balance and improve recovery outcomes in OUD treatment.^17^ A validated yoga module was proven feasible, safe, and potentially effective in alleviating withdrawal symptoms and cravings.^18^

This randomized clinical trial evaluated the efficacy of a 2-week validated yoga module as add-on therapy on primary outcomes: (1) HRV parameters reflecting autonomic regulation, and (2) time to recovery from opioid withdrawal symptoms in patients with OUD. Secondary objectives included determining the effects of yoga on anxiety, sleep quality, and pain, and assessing immediate pranayama effects on HRV.

## Methods

### Design and Setting

This early-stage randomized clinical trial was conducted in the Centre for Addiction Medicine (CAM) inpatient ward at the National Institute of Mental Health and Neurosciences (NIMHANS), a tertiary health care center in Bengaluru, India, between April 30, 2023 and March 31, 2024. The study was registered under the Clinical Trial Registry of India and was approved by the NIMHANS Institutional Ethics Committee. The trial protocol is available in Supplement 1. This study followed the Consolidated Standards of Reporting Trials (CONSORT) reporting guideline.

### Recruitment

Participants were recruited from the CAM inpatient ward. Written informed consent was obtained after explaining the study purpose to participants, ensuring confidentiality, and emphasizing voluntary participation.

### Eligibility Criteria

Adults aged 18 to 50 years with psychiatrist-diagnosed OUD, experiencing mild to moderate withdrawal symptoms based on Clinical Opiate Withdrawal Scale (COWS) scores (ranging from 4 to 24), and admitted to the CAM inpatient ward were included in the study. Exclusion criteria included patients with severe withdrawal (COWS score >25), comorbid neurological conditions affecting autonomic function (eg, traumatic brain injury, seizure disorders, and autonomic neuropathy), severe psychiatric conditions interfering with study participation (eg, active psychosis and severe cognitive impairment), prescribed opioid use for pain management, concurrent substance use disorders excluding nicotine, and prior exposure to specific yoga or other mind-body practices for OUD within 6 months.

### Study Procedures

#### Intervention: Yoga Add-On Therapy Group

Participants randomized to the yoga add-on therapy (YAT) group received 10 supervised yoga sessions for 14 days as add-on to treatment as usual. More than 90% of participants completed a minimum of 8 sessions. Each 45-minute session was conducted daily at 11:00 am in the designated yoga hall or at bedside to accommodate individual needs by a certified yoga therapist. The intervention employed a validated yoga module specifically designed for managing OUD withdrawal symptoms.^18^

The module comprised 5 standardized components: (1) relaxation practices, (2) gentle postures performed mindfully (asanas), (3) sectional breathing techniques for breath regulation, (4) gentle stimulation followed by relaxation through slow breathing practices (pranayama), and (5) guided relaxation with positive affirmations (abbreviated yoga nidra). Details are provided in eTable 1 in Supplement 2. These components were systematically selected to target key withdrawal symptoms: restlessness, anxiety, and sympathetic nervous system hyperactivity.^18^ Yoga practice proficiency was assessed at weeks 1 and 2 using the Yoga Performance Assessment (YPA) scale.

#### Control: Treatment-as-Usual Group

The control group received treatment as usual (TAU). This consisted solely of the standard pharmacological care and routine clinical management provided at the center.

#### Standard Pharmacological Care (All Participants)

All participants in both the YAT and TAU groups received buprenorphine as part of their standard care (eMethods 1 in Supplement 2). The specific dosage for each participant was determined and regulated by their treating psychiatrist based on daily assessments of withdrawal severity using the COWS.

#### Randomization and Allocation Concealment

The REDCap (Research Electronic Data Capture) platform,^19^ hosted at NIMHANS, was used for the collection and management of all study data. An independent statistician created the allocation sequence using REDCap’s randomization module, which employed variable block sizes (4 and 6) for a 1:1 randomization ratio. Allocation concealment was maintained by REDCap’s real-time reveal feature; after consent and baseline assessment, a research coordinator triggered the system to assign the participant to a group. This process was immediate, logged, and irreversible, preventing any influence on group assignment by study staff or assessors.

#### Assessment and Blinding

Outcome assessors and the data analyst were blinded to group allocation. Yoga instructors could not be blinded due to the nature of intervention delivery. Blinding was maintained through several procedural and system-level safeguards, including separating assessors from intervention delivery, instructing participants and assessors not to discuss allocation, scheduling assessments at different times from yoga sessions, and configuring assessors’ REDCap user rights to deny access to the allocation field.

The success of this procedure was formally evaluated after the trial. A blinding index questionnaire confirmed that assessors’ guesses of group allocation were no better than chance (54% accuracy; Cohen κ = 0.08), indicating that effective blinding was maintained.

### Outcome Measures

The preregistered primary outcomes were withdrawal symptoms severity (based on COWS scores) and HRV. COWS scores were recorded daily for 2 weeks. Recovery from withdrawal was operationalized as the first day a participant’s COWS score dropped below 4 and remained below this level thereafter. A cutoff of less than 4 was chosen because COWS scores of 5 or greater indicate at least mild withdrawal; thus, scores less than 4 reflect stabilization into minimal or asymptomatic state. Requiring scores to remain below this threshold reduced risk of misclassifying transient fluctuations as recovery. Given nonlinear withdrawal symptom trajectory, time to recovery was analyzed using Cox proportional hazards modeling, which captures stabilization timing variability and provides interpretable group difference estimates while adjusting for cumulative buprenorphine dosage.

HRV assessments were conducted on days 1 and 15 between 7:00 am and 8:00 am under standardized conditions. Participants fasted overnight and abstained from stimulants (eg, tea and coffee) before the assessment. A standardized 30-minute physiological recording protocol was implemented after a 10-minute supine rest. The YAT group protocol comprised the following exercises while sitting comfortably: (1) a 10-minute resting baseline, (2) 5-minute left-nostril breathing (LNB), (3) 5-minute humming breath (bhramari pranayama [BHM]), and (4) a 10-minute recovery period. The TAU group followed an identical setting and timeline but engaged in quiet breath observation during pranayama segments.

Throughout sessions, 2-channel electrocardiograph (ECG; 256 Hz), respiration (25.6 Hz), and 3-axis accelerometry data were acquired using an eq02+ LifeMonitor (Equivital). Data were processed in Python (Python Software Foundation) using the NeuroKit2 package. Recordings were segmented into epochs corresponding to each protocol phase (baseline, LNB, BHM, and recovery). Within each epoch, ECG signals were cleaned, R-peaks identified, R-R intervals (the time intervals between successive R-peaks in the electrocardiogram) calculated, and respiration metrics (ie, rate, amplitude, and count) derived. Finally, frequency-domain HRV indices were computed using multitaper power spectral density estimation (eMethods 2 in Supplement 2).

Secondary outcomes, measured at baseline (day 1) and postintervention (day 15), included validated clinical scales for anxiety assessment (Hamilton Anxiety Rating Scale [HAM-A]),^20^ pain evaluation (Brief Pain Inventory [BPI]),^21^ and sleep latency (patient self-reports of estimated time to fall asleep using sleep diaries).

### Sample Size

Sample size was determined a priori using G*Power, version 3.1 (University of Düsseldorf). On the basis of previous research examining HRV changes in people with OUD,^10^ we anticipated a medium effect size (Cohen *d* = 0.40) for primary HRV outcomes. To detect a significant group-by-time interaction in repeated measures analysis of variance with an α of .05 and power of 0.80, a minimum of 52 participants were required for 2 groups across 2 measurement points. Accounting for anticipated 15% attrition, the recruitment target was 60 participants randomized to 2 groups (30 per group).

**Figure 1.**
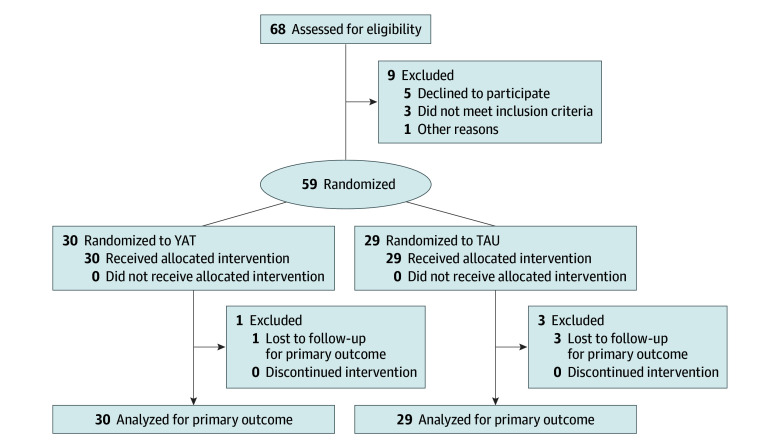
Flow Diagram TAU indicates treatment as usual; YAT, yoga as an add-on therapy.

All randomized participants were included in intent-to-treat analysis using linear mixed models, which appropriately handle missing data from participant attrition. Complete participant flow is detailed in the CONSORT diagram (Figure 1).

### Statistical Analysis

All statistical analyses were conducted using R, version 4.4.1 (R Core Team). Baseline demographic and clinical characteristics between YAT and TAU groups were compared using independent samples *t* tests for continuous variables and χ^2^ tests for categorical variables.

The primary outcome of HRV was analyzed using linear mixed-effects models (LMM) using the lme4 package, which are robust for handling repeated measures and missing data. Separate LMMs were constructed for each outcome (HRV indices, HAM-A anxiety scores, sleep latency, and pain scores). Models included time (day 1 vs day 15), group (YAT vs TAU), and time-by-group interaction as fixed effects. Random intercepts for each participant accounted for individual baseline variability. Skewed outcome variables, such as low frequency/high frequency (LF/HF) ratio, were log-transformed to meet model assumptions. Effect size magnitudes were calculated as partial omega-squared (ω^2^) using the effectsize package.

The co–primary outcome of time to recovery from opioid withdrawal was assessed using survival analysis. Recovery was defined as achieving stable minimal withdrawal: the first day a participant’s COWS score dropped below 4 and remained below 4 for subsequent observations. Time to this event was calculated in days from baseline. Participants not achieving stable recovery by final assessment were censored at the last observation day. Cox proportional hazards modeling using the survival package evaluated group allocation effects on recovery rate while controlling for cumulative buprenorphine dosage.

Mediation analysis tested whether changes in parasympathetic activity (M), indexed as ΔHF (day 15 minus day 1), mediated yoga’s effect (X) on withdrawal stabilization (Y). Total group effect on time to stabilization was estimated with Cox proportional hazards modeling. The a-path regressed ΔHF on group using linear regression, and b- and c′-paths were estimated with Cox modeling including both group and ΔHF, with ΔHF as a continuous variable expressed as log-hazards. Indirect effect (a × b) was calculated as the product of linear regression coefficient and log-hazard, then exponentiated to obtain indirect hazard ratio (HR). Percentile 95% CIs for indirect, direct, and total effects, as well as proportion mediated, were derived using 2000 cluster bootstraps. For transparency and reproducibility, a script implementing this statistical method is provided in eMethods 3 in Supplement 2.

For primary analyses, 2-sided *P* < .025 was considered statistically significant, with correction for 2 prespecified outcomes (COWS scores and HRV). For secondary analyses, the threshold was 2-sided *P* < .05. Effect sizes with 95% CIs are reported.

## Results

### Participant Characteristics

Among 68 patients screened, 9 were excluded, including 1 YAT participant and 3 TAU participants who withdrew from the study after randomization (Figure 1). The final randomized sample of 59 participants (59 male [100%]; mean [SD] age, 25.6 [3.9] years) was adequately powered for primary analyses, with 30 participants in the YAT group and 29 in the TAU group.

At baseline, YAT and TAU groups were well matched with no statistically significant differences on key demographic or clinical characteristics, except for age of first substance use and first opioid use (Table 1). Buprenorphine dosages (initial, mean daily, and stabilization) did not differ significantly between groups (Table 1).

**Table 1.** Clinical and Demographic Characteristics

Characteristic	TAU (n = 29)	YAT (n = 30)	Total (N = 59)
Age, mean (SD), y	24.90 (4.33)	26.27 (3.33)	25.59 (3.88)
Male sex, No. (%)	29 (100.0)	30 (100.0)	59 (100.0)
BMI, mean (SD)	18.46 (2.23)	20.00 (4.10)	19.26 (3.39)
10th Grade standard education or less, No. (%)	23 (79.3)	16 (53.3)	39 (66.1)
Unemployed, No. (%)	22 (75.9)	20 (66.7)	42 (71.2)
Married, No. (%)	4 (13.8)	10 (33.3)	14 (23.7)
Age at first substance use, mean (SD), y	13.97 (2.67)	16.70 (4.20)	15.36 (3.76)
Age at first opioid use, mean (SD), y	19.86 (3.89)	22.57 (3.82)	21.24 (4.06)
First buprenorphine dose, mean (SD), mg	1.18 (1.40)	1.02 (2.18)	1.09 (1.84)
Buprenorphine dosage at withdrawal stabilization, mean (SD), mg	3.65 (2.27)	2.67 (4.47)	3.08 (3.71)
Buprenorphine dosage, mean (SD), mg	3.15 (1.96)	3.8 (4.61)	3.53 (3.5)
Primary opioid category, No. (%)			
Tapentadol	23 (79.3)	24 (80.0)	47 (79.7)
Semisynthetic opioids (eg, heroin)	5 (17.2)	4 (13.3)	9 (15.3)
Other synthetic opioids	1 (3.4)	2 (6.7)	3 (5.1)
Frequency of daily use during past month, No. (%)	26 (89.7)	28 (93.3)	54 (91.5)
Last opioid use within 72 h, No. (%)	20 (69.0)	27 (90.0)	47 (79.7)
Opioid withdrawal severity, mean COWS score (SD)	14.66 (4.39)	12.90 (3.94)	13.78 (4.23)

Abbreviations: BMI, body mass index (calculated as weight in kilograms divided by heigh in meters squared); COWS, Clinical Opiate Withdrawal Scale; NA, not applicable; TAU, treatment as usual; YAT, yoga as an add-on therapy.

### Primary Outcomes

#### Autonomic Function

**Figure 2.**
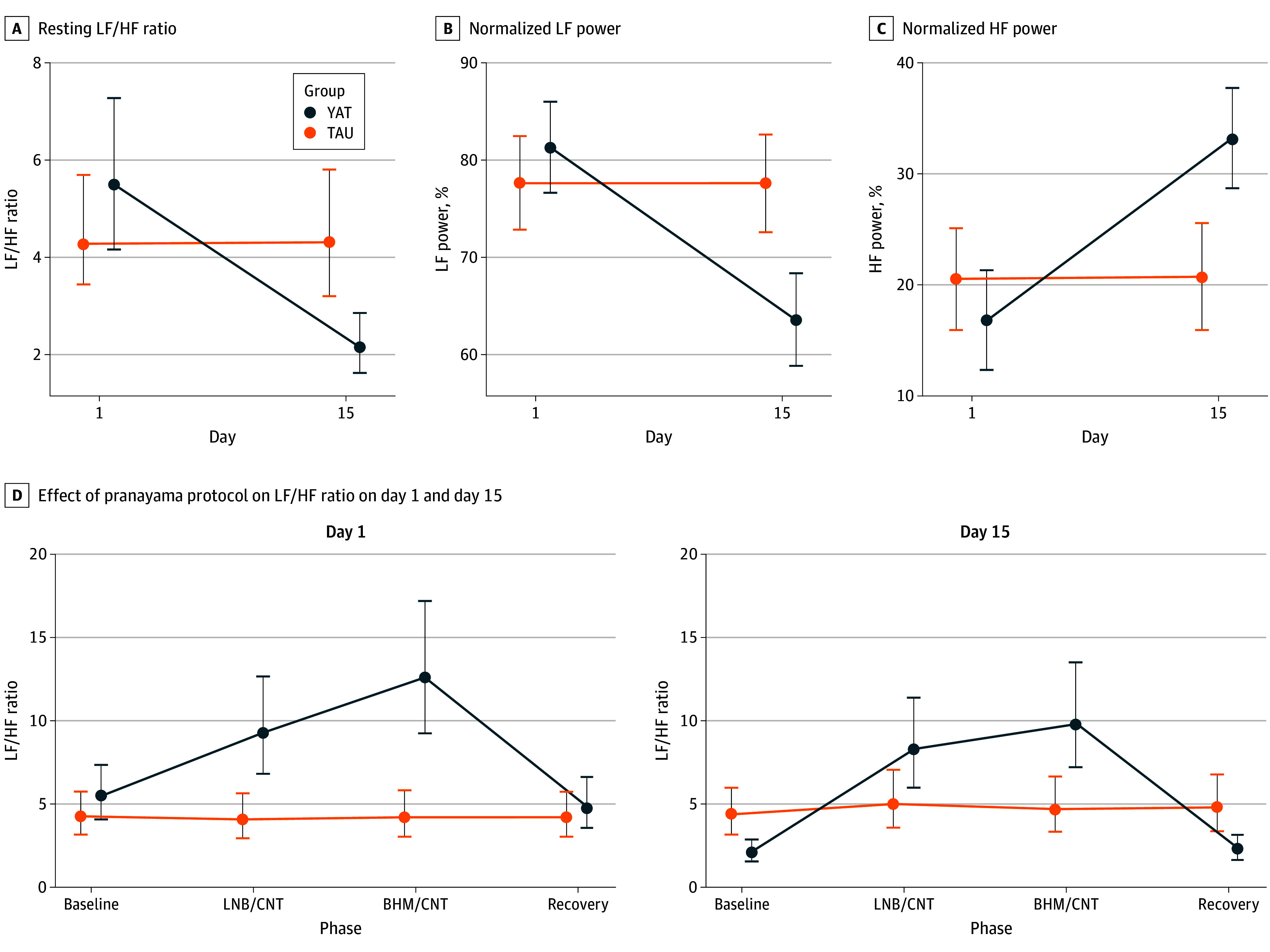
Heart Rate Variability Changes Between Yoga and Control Groups A-C, Panels show changes from baseline (day 1) to postintervention (day 15) in the resting phase low frequency/high frequency (LF/HF) ratio (A), normalized LF power (B), and normalized HF power (C). A significant group-by-time interaction effect was observed for all 3 measures (*P* < .001). D, Panel shows the immediate effect of the pranayama protocol on the LF/HF ratio on day 1 (left) and day 15 (right). Phases include baseline rest, left-nostril breathing (LNB)/control (CNT), bhramari pranayama (BHM)/CNT, and recovery. Points indicate estimated marginal means; error bars, 95% CIs. TAU indicates treatment-as-usual group; YAT, yoga add-on therapy group.

Analysis of resting-state HRV parameters revealed significant group-by-time interactions, indicating that the yoga intervention led to superior improvements in autonomic regulation compared with TAU (Table 2; Figure 2). Specifically, the YAT group exhibited greater reductions in the LF/HF ratio (*P* < .001; ω^2^ = 0.12, representing a medium effect) and normalized LF power (*P* < .001; ω^2^ = 0.16, representing a large effect), with greater increases in normalized HF power (*P* < .001; ω^2^ = 0.14, representing a large effect), reflecting a shift toward enhanced parasympathetic balance.

**Table 2.** Estimated Marginal Means and Group × Time Interaction Effects for Heart Rate Variability and Clinical Outcomes

Variable	EMM (95% CI)				Group × time interaction (95% CI)	T value	Partial ω^2^ (95% CI)^a^
	Day 1		Day 15				
	TAU	YAT	TAU	YAT			
LF power, nu	77.60 (72.87 to 82.33)	81.27 (76.63 to 85.92)	77.60 (72.65 to 82.55)	63.59 (58.87 to 68.30)	−17.69 (−25.29 to −10.09)	−4.61^b^	0.16 (0.05 to 0.28)
HF power, nu	20.64 (16.16 to 25.12)	16.96 (12.55 to 21.37)	20.83 (16.13 to 25.53)	33.18 (28.71 to 37.65)	16.03 (8.74 to 23.32)	4.36^b^	0.14 (0.04 to 0.26)
LF/HF ratio	4.26 (3.21 to 5.67)	5.48 (4.14 to 7.25)	4.29 (3.18 to 5.79)	2.14 (1.61 to 2.84)	−0.95 (−1.41 to −0.48)	−4.04^b^	0.12 (0.03 to 0.24)
HAM-A score	16.43 (15.25 to 17.61)	17.21 (16.05 to 18.36)	8.16 (6.94 to 9.38)	1.93 (0.77 to 3.09)	−7.01 (−9.11 to −4.91)	−6.63^b^	0.28 (0.15 to 0.41)
Sleep latency, min	152.50 (128.88 to 176.12)	152.59 (129.38 to 175.80)	82.89 (58.39 to 107.39)	21.90 (−1.31 to 45.11)	−61.08 (−105.72 to −16.44)	−2.71^c^	0.06 (0.00 to 0.16)
Average pain severity in 24 h, VAS score	34.86 (28.54 to 41.17)	33.76 (27.56 to 39.96)	17.94 (11.42 to 24.47)	1.00 (−5.20 to 7.20)	−15.85 (−26.38 to −5.31)	−2.98^c^	0.07 (0.01 to 0.18)

Abbreviations: EMM, estimated marginal means; HAM-A, Hamilton Anxiety Rating Scale; HF, high frequency; LF, low frequency; nu, normal units; TAU, treatment as usual; VAS, visual analog scale; YAT, yoga as an add-on therapy.

^a^ Partial ω^2^ effect sizes: small effect ≈ 0.01, medium effect ≈ 0.06, large effect ≈ 0.14.

^b^ *P* < .001.

^c^ *P* < .01.

#### Opioid Withdrawal Stabilization

**Figure 3.**
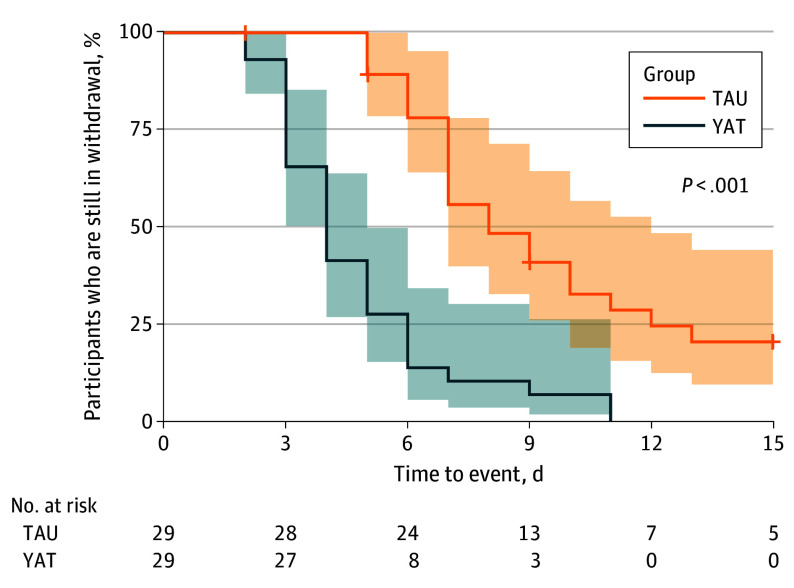
Time to Withdrawal Stabilization for the Yoga vs Control Groups Survival curves comparing the time to withdrawal stabilization for the yoga as an add-on therapy (YAT) and treatment-as-usual (TAU) groups. The y-axis represents the percentage of participants who have not yet achieved stabilization (ie, are still in withdrawal). The steeper decline of the YAT curve demonstrates a significantly faster time to recovery compared with the TAU group (*P* < .001 from log-rank test). Shaded areas indicate 95% CIs. The table below the plot shows the number of participants remaining at risk (ie, not yet stabilized) in each group at key time points.

Survival analysis demonstrated that the YAT group achieved withdrawal stabilization significantly faster than the TAU group. YAT participants had a higher rate of reaching the recovery milestone (defined as stable COWS score <4) compared with TAU participants (HR, 4.40; 95% CI, 2.40-8.07; *P* < .001). After accounting for cumulative buprenorphine dosage, median time to withdrawal stabilization was 5 days (95% CI, 4-6 days) in the YAT group compared with 9 days (95% CI, 7-13 days) in the TAU group (Figure 3). This accelerated recovery occurred despite no significant difference in cumulative buprenorphine dosage between groups.

### Secondary Clinical Outcomes

Beneficial effects of yoga were further supported by significant improvements in secondary clinical outcomes (Table 2; eFigure 1 in Supplement 2). A large, significant group-by-time interaction was observed for anxiety, with the YAT group showing substantially greater HAM-A score reduction compared with the TAU group (*P* < .001; ω^2^ = 0.28). Significant interaction effects indicated greater improvements in sleep latency in the YAT group (approximately 61 minutes more reduction than in the TAU group; *P* = .008; ω^2^ = 0.06) and average pain perception (BPI average pain severity [0-10 scale], converted to a visual analog scale [VAS] score of 0-100 for analysis consistency) (*P* = .004; ω^2^ = 0.07).

### Process Measures

To assess intervention fidelity, a qualified yoga therapist rated each participant’s performance using the YPA on days 7 and 15. Therapist-rated scores increased significantly from the first to the second week, confirming participants’ ability to correctly perform the yoga module improved over time (eFigure 2 in Supplement 2).

Immediate physiological effects of pranayama practices were analyzed during 30-minute recording sessions on days 1 and 15 (Figure 2). YAT participants significantly reduced mean respiration rate from baseline of approximately 21 breaths/min to approximately 12 breaths/min during active LNB and BHM phases. In contrast, the respiratory rate in the TAU group remained stable at approximately 20 breaths/min throughout sessions (eTable 1 and eFigure 3A in Supplement 2). Results indicate consistent transient sympathetic activation during pranayama, followed by enhanced parasympathetic rebound during recovery (Figure 2; eFigure 3B-C in Supplement 2).

Mediation analysis showed increases in parasympathetic activity (ΔHF power) partially accounted for yoga’s effect on withdrawal recovery. The indirect effect was significant (HR, 1.38; 95% CI, 1.10-2.03), explaining 23% (95% CI, 9%-36%) of total effect. The direct effect remained robust (HR, 2.98; 95% CI, 2.56-7.50), suggesting mechanisms beyond HRV improvement also contributed. Mediation effects through LF power and LF/HF ratio were not significant.

## Discussion

This early-stage randomized clinical trial provides evidence that yoga, as adjuvant to standard buprenorphine treatment, improves opioid withdrawal outcomes. Our primary finding was accelerated recovery, with the YAT group achieving minimal withdrawal symptoms at a rate 4.4 times higher than the TAU group. This difference in recovery rate translated to reduced median stabilization time from 9 to 5 days, underpinned by improvements across physiological (HRV), psychological (anxiety), and symptomatic (sleep and pain) domains.

### Primary Outcomes

The implications of this stabilization are notable. Baillet et al^19^ found that craving trajectories during the first 14 days were associated with long-term substance use outcomes. By shortening the withdrawal period when relapse risk and dropout rates are highest, yoga may influence these trajectories, potentially improving long-term retention and outcomes.

Our survival analysis, controlling for cumulative buprenorphine dosage, demonstrated that yoga’s effects were independent of medication optimization, providing benefit beyond pharmacological care. The basis likely lies in impact of yoga on the autonomic nervous system. Cardiac vagal tone decreases during opioid withdrawal, indicating a need for parasympathetic interventions.^10^ While sympatholytic medications such as clonidine are used, they may not address reduced parasympathetic tone.^20^ Our findings align with those of Tyagi and Cohen,^21^ who found that yoga practices increase parasympathetic activity (HF power), addressing this gap.

Although this trial lacked an active control, observed HRV changes point to autonomic regulation as one pathway through which yoga facilitates withdrawal recovery. Mediation analysis showed increases in parasympathetic activity (HF power) significantly mediated yoga’s effect, accounting for 23% of the total effect. The direct effect remained significant, indicating that additional processes, such as anxiety, sleep, and pain improvements, likely interact with or arise from autonomic changes to support recovery. Mediation through LF power and LF/HF ratio was not significant, highlighting parasympathetic enhancement’s specificity as a mechanistic pathway.

Our results align with research on other contemplative practices, such as autonomic improvements during Zen meditation in substance use disorder.^22^ However, our study extends this work by demonstrating autonomic recalibration with effect sizes and establishing HRV improvements’ significance through their link to withdrawal recovery. These results suggest that while contemplative practices may share neurobiological pathways, structured and repeated practice is necessary to achieve addiction recovery outcomes.

### The Autonomic Rehabilitation Effect

Our immediate pranayama assessments revealed a paradoxical response. Respiratory rate slowed from approximately 20 breaths/min to approximately 12 breaths/min (0.2 Hz), which is typically associated with greater parasympathetic HF power; however, we observed the opposite: a transient increase in LF/HF ratio and LF power, with reduced HF power. Because breathing frequency remained within the HF band, this finding likely reflects genuine sympathetic activation rather than artifact.

This HF power suppression during pranayama has 2 explanations. First, paced rhythmic breathing reduces natural respiratory variability, attenuating respiratory sinus arrhythmia.^22,23^ Second, the cognitive and physiological demands of breath regulation impose additional vagal withdrawal.^23,24^ Studies confirm some pranayama techniques acutely suppress HF power,^22,23,25,26^ whereas others, such as alternate-nostril breathing or om chanting, can enhance HF-HRV, especially with practice experience.^27,28^ These findings suggest that transient sympathetic activation is more prominent in novices or clinical populations and may attenuate with sustained practice, allowing parasympathetic reset over time.

In individuals with OUD, already marked by sympathetic hyperarousal, such perturbations may act as autonomic stress tests. Importantly, activation was consistently followed by recovery, and by day 15, the yoga group showed significantly lower LF/HF ratios and higher HF power at rest compared with baseline. This pattern supports autonomic rehabilitation, where repeated suppression-rebound cycles recalibrate vagal function, enhance baroreflex sensitivity, and counter noradrenergic hyperactivity. Over time, these adaptations may consolidate into improved autonomic balance and resilience.

### Secondary Outcomes

Beyond primary findings, yoga produced benefits. Anxiety was reduced, with effect size (ω^2^ = 0.28) comparable to anxiolytic interventions.^24^ This finding is important, as anxiety during recovery is a driver of craving and relapse. Additionally, participants demonstrated a 61-minute reduction in sleep latency and improvements in pain perception, extending findings from chronic pain populations^24,25^ to withdrawal setting. Although effect sizes for sleep (ω^2^ = 0.06) and pain (ω^2^ = 0.07) were medium, they represent benefits that likely contributed to recovery.

### Strengths and Limitations

This study has some strengths. The absence of an active control limits causal inference, but findings argue against purely placebo effects. Objective HRV improvements, including transient sympathetic activation during pranayama followed by parasympathetic recovery, align with yoga’s known mechanisms.^25,26,29^ Mediation analysis showed that HF-HRV increases significantly mediated accelerated recovery, supporting mechanism-based interpretation. All participants received standardized buprenorphine withdrawal following established guideline principles for opioid withdrawal management (aligned with National AIDS Control Organisation recommendations),^30^ with physician training and daily COWS monitoring ensuring adherence. Cumulative buprenorphine dosage was included as a covariate, reducing likelihood that medication variability explained group differences.

Limitations included a single-center design, short intervention duration, and male-predominant sample, restricting generalizability. Gender imbalance reflects local trends of opioid misuse predominantly affecting men,^31^ yet underscores the need for multisite trials with purposive female recruitment. Future studies should examine acceptability and efficacy of yoga across diverse populations and compare it with other evidence-based psychosocial interventions. Although tapentadol was the primary opioid being misused, sympathetic hyperactivation characterizes withdrawal across agents, including morphine and fentanyl.^3,32^ Evidence has shown that opioid withdrawal broadly impairs vagal tone,^12^ HRV reductions correlate with severity,^13^ and chronic exposure blunts HF power.^10^ Thus, the sympathovagal benefits of yoga likely extend to other opioids, but replication in diverse settings and in fentanyl-dependent populations is needed. Additionally, feasibility in community treatment contexts remains to be established. Future research should include larger, multisite trials with diverse populations, longer interventions, active controls, and dose-response studies. Comparing effectiveness across different opioid types and optimal intervention timing would further inform clinical implementation.

## Conclusion

In this randomized clinical trial, adjuvant yoga therapy significantly accelerated opioid withdrawal recovery while addressing autonomic dysregulation. The concurrent physiological, psychological, and symptomatic improvements suggest that yoga may restore core regulatory processes beyond symptom management. By targeting parasympathetic restoration, yoga may fill a critical therapeutic gap in standard OUD care, supporting integration into withdrawal protocols as a neurobiologically informed intervention with potential economic benefits.

## Notes

Supplement 1.
Trial Protocol

Supplement 2. **eMethods 1.** Detailed HRV Preprocessing Protocol **eMethods 2.** Standard Buprenorphine Protocol **eMethods 3.** Mediation Analysis Script **eTable 1.** Key Physiological Parameters by Group and Time **eTable 2.** Yoga Module for Opioid Withdrawal - Practice Details and Rationale **eFigure 1.** Linear Mixed-Effects Model Plots Showing Changes in Clinical Outcome Measures Between Yoga and Control Groups **eFigure 2.** Yoga Performance Assessment (YPA) Scores at Day 7 and Day 15 **eFigure 3.** Linear Mixed-Effects Model Plots Showing Changes in Heart Rate Variability (HRV) During the Pranayama Protocol Between Yoga and Control Groups

Supplement 3.
Data Sharing Statement
