# Supplementary material for: Yoga for Opioid Withdrawal and Autonomic Regulation: A Randomized Clinical Trial
Source: JAMA Psychiatry. 2026 Jan 7;83(3):238–46. doi: 10.1001/jamapsychiatry.2025.3863 (PMC12780978; doi:10.1001/jamapsychiatry.2025.3863)
Supplement: Supplement 1. — Trial Protocol [file jamapsychiatry-e253863-s001.pdf]

NATIONAL INSTITUTE OF MENTAL HEALTH AND NEURO SCIENCES
(INSTITUTE OF NATIONAL IMPORTANCE), BANGALORE - 560 029

Date: 20.01.2023

From
Dr. Suddala Goutham,
Student of MSc Yoga Therapy (Mental health and Neuroscience),
Department of Integrative Medicine, NIMHANS Bengaluru.

To
Chairperson/Member Secretary
NIMHANS Ethics Committee
National Institute of Mental Health and Neurosciences
Bangalore-560029

"Through Proper Channel"

Respected Sir Madam,

Sub: Ethical clearance for the M.Sc Thesis Protocol titled "Role of Adjuvant Yoga Therapy in Managing Withdrawal Symptoms of Patients with Opioid Use Disorder"

I am herewith submitting 1 + 1 (Hard Copy) of the MSc Thesis/ dissertation Protocol titled "Role of Adjuvant Yoga Therapy in Managing Withdrawal Symptoms of Patients with Opioid Use Disorder" for consideration by the Departmental Ethics Sub-committee. Theprotocol has to undergo scientific review and approval at the Departmental level.

Thanking you,

Yours sincerely,

Dr. SUDDALA GOUTHAM
Name & Signature of the student
Encl. As above

Remarks of the Guide with name, signature and seal

R & F

Professor & Head
Department of Integrative [illegible]
NIMHANS
Bangalore [illegible]

Remarks of HOD with Name, Signature & Seal:

**Response to Queries Raised by the Ethics Committee**

| Sl. No | Comments Raised | Response to Comment | Page no. where modifications are made. |
|---|---|---|---|
| 1. | In Summary sheet – Item 8 – As there is an intervention involved in the study it can't be considered as "less than minimal risk". The investigators should choose the category appropriately, and mention measures taken to minimize the risk. | Appropriate category mentioned with measures taken to minimize the risk. | Highlighted by underlining the text on page no. 12 |
| 2. | ICF and the ethics summary page to be checked carefully and edited in keeping with the suggestions given during the meeting. | ICF and ethics summary page have been thoroughly checked and edited. | Highlighted by underlining the text on page no. 10,11,12 |
| 3. | In ICF, under the section "who is organizing the study", the department and the institute should be mentioned. | The department and the institute has been mentioned under the section "who is organising the study", in ICF. | Highlighted by underlining the text on page no. 12 |
| 4. | The exclusion criteria for the study is not clear and has to be specified, including details of medications to be taken or avoided. | In the study, we are not excluding patients who are taking medications other than Buprenorphine, but we will document all the prescribed drugs taken by the patients in both groups. | The changes are mentioned under the section of study design, highlighted by underlining the text on page no. 20 |

**Checklist of Documents for dissertation/thesis**

**(Required to be submitted to Ethics Sub-committee)**

| **Documents** (1+1 Hard Copy + PDF e-version by e-mail with all the duly filled-in IEC formats including cover letter) | **Remarks** (Tick the appropriate) |
|---|---|
| 1. Cover letter for the project proposal ("Through Proper Channel" | Yes |
| 2. Summary Sheet of the Thesis/dissertation Protocol | Yes |
| 3. Research Project proposal submitted for Ethical Clearance as per the NIMHANS Ethics Committee guidelines | Yes |
| i. Consent form duly signed by the student / collaborators. (Duly signed Attestation & Declaration Form) | Yes |
| ii. Consent of the concerned Head of the department | Yes |
| iii. Authorization/ sanctioning letter (finance sanction) from sponsoring agency | No |
| iv. Informed Consent form as per the guidelines of NIMHANS IEC | Yes |
| v. Consent form for carrying out the required investigations from the concerned Heads of Department(s), if applicable | Yes |
| vi. Undertaking by the student | Yes |

**SUMMARY SHEET OF THE THESIS PROTOCOL SUBMITTED TO THE DEPARTMENTAL ETHICS SUB-COMMITTEE, NIMHANS**

**Submitted on: 20-01-2022**

| | | |
|---|---|---|
| 1. | **Title and duration of the project** | Role of Adjuvant Yoga Therapy in Managing Withdrawal Symptoms of Patients with Opioid Use Disorder Duration: 1 year |
| 2. | **Student and Department** | Dr. Suddala Goutham<br><br>Student of MSc Yoga Therapy(Mental health and Neuroscience)<br><br>Department of Integrative Medicine, NIMHANS<br><br>Bengaluru. |
| 3. | **Funded or Non funded project? If yes, name of the funding agency (Govt, Private, Foreign)**<br><br>**OR**<br><br>**Is the project being submitted for funding?** | Funded by DBT Wellcome India Alliance, PI – Dr Hemant Bhargav |
| 4. | **Are human subjects involved in the study? IF yes, mention type of participants (patients, relatives, professionals etc.** | Yes<br><br>Patients |
| 5. | **Does the study involve healthy volunteers?** | No |
| 6. | **Does the study involve a vulnerable population (Children, Pregnant women, persons with disabilities, persons with mental illness, etc.)?** | Yes<br><br>Persons with Mental illness |
| 7. | **Study Design (Describe briefly)** | Two-arm prospective Randomized controlled clinical Trial.<br><br>The first arm will receive Yoga + treatment as usual<br><br>The second arm will receive only treatment as usual |

| | | |
|---|---|---|
| 8. | **Procedures and risks: list the procedures carried out and the possible risk (classified as less than minimal, minimal, low or high)** | <u>Procedures</u><br>1) Breathing exercises<br>2) Physical postures (asanas)<br>3) Regulated breath practices (pranayama)<br>4) Relaxation techniques<br><br><u>Risk</u><br><u>The risk is less than minimal</u> |
| 9. | **Detail the measures taken for reducing the risk** | The risk is less than minimal but if any discomfort experienced the practice will be immediately stopped and relaxation will be given. To avoid any minimal risk of strain, every practice will be followed by a brief relaxation. |
| 10. | **Does the study involve biological specimens? If yes, list the type of specimens and the amount** | No |
| 11. | **Does the research/study involve:**<br>• **Human exposure to radioactive agents?**<br>• **Human exposure to infectious agents?**<br>• **Investigational new drug?**<br>• **Investigational new device?**<br>• **New treatment regimen?**<br>• **Use of new vaccines?**<br>• **Observation of public behavior?**<br>• **Fetal tissue or abortus?**<br>• **Pathological or diagnostic clinical specimen only? (Mention source………….)**<br>• **Existing data available via public archives source? (Specify………………….)**<br>• **Existing data available from co-investigator?** | <br>No<br>No<br>No<br>No<br>No<br>No<br>No<br>No<br>No<br>No<br>No |
| 12. | **Is the informed consent form attached? If not, mention the justification** | Yes |
| 13. | **Does the informed consent form address the following?** | |

| | | |
|---|---|---|
| | **a) Provide adequate information about the title, purpose, procedures, & details of participation in a layperson's language to the participant?**<br>**b) Is the method of selection of subjects including random selection, if applicable is explained?**<br>**c) Are procedures including invasive procedures and possible risks adequately explained?**<br>**d) Are financial implications explained to the patient/legal guardian?**<br>**e) Are there separate ICF's for participants, legally acceptable representatives (LAR'S) and volunteers? If so, list**<br>**f) If subject is a minor, is there an appropriate assent form?**<br>**g) Is the course of action, in case, any abnormalities are detected during the investigation, clearly spelt out?**<br>**h) Is provision for the subject to opt out of the study made explicitly**<br>**i) Is the confidentiality of the subject's data assured**<br>**j) If major risks are involved, is the mechanism of treatment/compensation for any injury suffered (e.g., Insurance) clearly spelt out?**<br>**k) Are the contact details of the PI and investigators provided?**<br>**l) If the study involves a biological specimen, is the consent obtained only for the current study or for future research also?**<br>**Are the details of payments included?** | Yes<br>Yes<br>NA<br>NA<br>NA<br>NA<br>NA<br>NA<br>NA<br>Yes<br>NA<br>NA |
| 14. | **In case of Clinical Trials** | |
| | **a) Name of the drug (device) being investigated?** | **Therapeutic Procedures**:<br>Yoga module for substance use disorder during Acute withdrawal phase |
| | **b) Is the product currently in clinical use in India?** | Yes |
| | **c) Does the trial involve an investigational new drug** | No |
| | **d) Name and address of the manufacturer?** | NA |
| | **e) Is there a DCGI approval for the trial?** | NA |
| | **f) Is it a multi-centric trial? If yes, how many Indian and how many foreign centers are involved?** | No |

| | | |
|---|---|---|
| | **g) Does the drug have statutory approval for clinical use in the country of origin?** | NA |
| | **h) Does the study have a placebo arm? If does, what risk does it entail for the subjects? justify the use of the placebo** | No |
| | **i) Is any standard treatment withheld in any subject as a part of the study? If yes, provide justification for the same and assurance of patient safety** | No |
| | **j) Are the possible risks documented in the literature adequately explained in the ICF** | NA |
| | **k) Is the trial covered by an insurance scheme? (If yes, details)** | No |
| | **l) Are there any conflicts of interest for the investigators: e.g., remuneration paid to the investigator by the company, investigator's financial involvement in the company** | No |
| | **m) Implications of costs to the patient**<br>**n) Does the trial cover the patient's treatment cost?**<br>**o) Does the trial pay for the additional costs to the patient on account of his participation in the trial?**<br>**p) Is the patient/volunteer provided remuneration for participating in the trial?** | NA<br>Yes<br>No<br>No |
| 15. | **In the case of chart review / records-based studies, mention how the identity of the patient is de-linked and how confidentiality is maintained** | NA |
| 16. | **a. Does the research deal with sensitive aspects of the subject's behavior such as sexual behavior, alcohol use or illegal conduct such as drug use?**<br>**b. Are there any elements in the protocol that are likely to induce anxiety or distress to the subject (e.g., intrusive questionnaire, presentation of material that is unpleasant to the participant etc.)** | No<br>No |
| 17. | **Is there payment to participants? If yes, the details** | No |
| 18. | **Mention specific Ethical Issues involved in the proposed research (List & briefly describe)** | No ethical issues are anticipated in this study. |

| | | |
|---|---|---|
| 19. | **Whether the proposed study is a collaborative study? Yes/No**<br><br>**If yes, does the other institution have IERB? Yes/No**<br><br>**If yes, have you received that IERB approval? Yes/No** | No<br><br>NA<br><br>NA |
| 20. | **Is there a Bio-safety Department in the collaborative institute for disposing of biological samples in a scientific manner after carrying out an investigation?** | NA |
| 21. | **Indicate the biological samples collected for the proposed study (blood/CSF/tissue/muscle biopsy etc.) If Yes, how often?** | NA |
| | **Will the samples be stored for future research Yes/No** | NA |
| 22. | **Mention whether the sample size allows enough power to detect the difference/results expected from the investigation** | Yes |
| 23. | **Is there any conflict of interest?** | No |
| 24. | **Is there financial interest of (i) investigators or (ii) sponsors? If yes, provide details** | No |

# CONSENT FORM

**"Role of Adjuvant Yoga Therapy in Managing Withdrawal Symptoms of Patients with Opioid Use Disorder"**

**Centre**: National Institute of Mental Health and Neurosciences, Bengaluru.

I certify that I have disclosed all details about the study in terms easily understood by the subject. Your consent to participate in the above study is sought. You have the right to refuse consent or withdraw the same during any part of the study without giving any reason. In such an event, you will still receive the best possible alternative treatment, without any prejudice. If you have any doubts about the study, please feel free to clarify the same. Even during the study, you are free to contact any of the investigators for clarification if you so desire (Dr. Suddala Goutham, Student of MSc. Yoga Therapy (Mental Health and Neuroscience), Department of Integrative Medicine. Phone number: XXXXXXX301). All the information/data collected from you (participant) will be kept in strict confidence.

CONSENT BY PARTICIPANT

1. I confirm that I have read / the study and has been explained to me adequately and I have understood the information for the above study and had the opportunity to ask questions.
2. I hope to complete the study, but I understand that my participation is voluntary and that I am free to withdraw at any time, without giving a reason, and without my medical care or legal rights being affected.
3. I understand that my doctor will provide information about my progress, in confidence, to the related officers of the participating Institution. I understand that the information held by the Investigators and researchers and records might be used to follow up my health status.
4. I understand that the information will be used for medical research only and that I will not be identified in any way in the analysis and reporting of the results. I understand that sections of any of my medical notes may be looked at by the Sponsors or responsible individuals of the Institutional Ethics Committee (IEC), Regulatory authorities or Court, if necessary. I give permission for these individuals to have access to my records.
5. I understand what is involved in this study and agree to take part in the study for a period of 2 weeks.
6. I am aware that by subjecting myself to this research, I will have to give more time for assessments by the investigating team and that these assessments do not interfere with the benefits.

| Name of participant | Signature/ Thumb impression | Date |
|---|---|---|
| | | |

**PATIENT INFORMATION SHEET**

**Title of the Study**

**"Role of Adjuvant Yoga Therapy in Managing Withdrawal Symptoms of Patients with Opioid Use Disorder"**

## 1. Introduction

You are invited to take part in a research study. Before you decide it is important for you to understand why the research is being done and what it will involve; please take time to read the following information carefully and discuss it with friends and relatives if you wish. Ask us if there is anything that is not clear or if you would like more information. Take time to decide whether or not you wish to take part. You should not sign this form until you understand all of the information presented in the following pages and until all of your questions about the research have been answered to your satisfaction.

## 2. What is the purpose of the study?

This is a research study being conducted at the National Institute of Mental Health and Neurosciences, Bengaluru, to understand the impact of Yoga as an add-on therapy in recovery from withdrawal symptoms of opioid use disorder when compared to standard treatment alone.

## 3. Why am I being invited?

Being a patient of an Opioid Use Disorder and fulfilling the inclusion and exclusion criteria of the study, we consider you as an eligible candidate for the study.

## 4. Do I have to take part?

It is up to you to decide whether or not to take part. If you do decide to take part you will be given this information sheet to keep and be asked to sign a consent form later. If you agree to take part you are still free to withdraw at any time and without giving any reason. This will not affect the standard of care you receive.

## 5. What will happen to me if I take part?

If you agree to take part in this study, you will be one of the 60 subjects we plan to recruit in this study. You will then randomly be divided into either the Yoga + treatment as usual group or the Treatment as usual only group. According to the allocation you will be given intervention for 2 weeks. During the invention, a few subjective scales will be taken at baseline, after the completion of 2 weeks of intervention. The assessments include the Clinical Opiate Withdrawal Scale (COWS), HRV (Heart rate variability), Anxiety (HAM-A), Brief Pain Inventory & Sleep latency. These assessments will give help us understand the change in symptoms, autonomic function of heart, sleep latency, pain levels. If you are in Yoga + treatment group, you will be practicing a yoga module for substance use disorder during acute withdrawal phase. No change will be made in the Psychiatric medications already being taken by you.

## 6. What investigations or treatments will be conducted in this study?

Yoga module for substance use disorder during acute withdrawal phase. There will be no drug tested.

**7. Will I benefit from this study?**

Your participation will help us understand the impact of yoga on early recovery from withdrawal symptoms. The results of the research may provide benefits to society in terms of the advancement of therapeutic benefits to future patients.

**8. Risks – What are the risks that I am likely to face if I participate in this study? What if something goes wrong?**

According to the previous studies which included the Yoga module there was no report of any major side effects or risks reported by the participants. There is minimal risk of strain, but if any discomfort experienced the practice will be immediately stopped and relaxation will be given. To avoid any minimal risk of strain, every practice will be followed by a brief relaxation.

**9. How will my confidentiality be protected?**

All your information will be kept confidential but any of your medical records may be inspected by the Investigator of the study for the purpose of analyzing the results. They may also be looked at by members of the Institutional Ethics Committee and by Regulatory authorities/courts to check that the study is being carried out correctly. Your name, however, will not be made public and any sensitive matter regarding your state of health will be kept confidential.

**10. What if I don't want to participate in this study, or I want to withdraw later?**

Yes, you have the right to refuse consent or withdraw the same during any part of the study without giving any reason.

**11. What happens with the data collected / results / my samples?**

The results of this trial will be published in leading medical journals so that other doctors and researchers can benefit from the results. You can ask your investigating physician for a copy of the publication. If published, your identity and personal details will be kept strictly confidential. No named information about you will be published in any of the trial reports.

**12. Who is organizing the study?**

This is funded by DBT Wellcome India Alliance, it is an MSc thesis under the Department of Integrative Medicine, NIMHANS, Bangalore.

**13. Who has reviewed this study?**

This study has been reviewed by Human Ethics Committee for Research in AYUSH and Integrative Medicine, NIMHANS, Bangalore.

**14. Whom should I contact for more information?**

If desirous of any relevant information at any stage of the clinical trial, you may feel free to ask your investigating physician for that information. You would be given a copy of the information sheet and a signed consent form.

Contact address of the Student :
Dr. Suddala Goutham (BNYS),
Student of MSc. Yoga Therapy (Mental Health and Neuroscience)
Department of Integrative Medicine, NIMHANS, Bengaluru;
Ph.no.XXXXXXXXX301; email: gouthamreddy148@gmail.com.

## Undertaking

I certify that I have disclosed all details about the study in terms easily understood by the subject. Your consent to participate in the above study is sought. You have the right to refuse consent or withdraw the same during any part of the study without giving any reason. In such an event, you will still receive the best possible alternative treatment, without any prejudice. If you have any doubts about the study, please feel free to clarify the same.
Even during the study, you are free to contact any of the investigators for clarification if you so desire (Dr. Suddala Goutham, Integrative department, Mob no: 8332924301). All the information/data collected from you (participant) will be kept in strict confidence.

## Certificate of Consent

I have read the participant information sheet/participant information sheet has been read out to me. I have been informed about the procedures of the study. The possible risks too have been explained to me as stated in the Participant Information Sheet. I have had the opportunity to ask questions about it and any questions that I have asked have been answered to my satisfaction I am aware that I have the right to refuse my consent or withdraw it at any time during the study without adversely affecting my treatment. I am aware that by subjecting myself to this research, I will have to give more time for assessments by the investigating team and that these assessments do not interfere with the benefits.

I……………………………………………….., the undersigned, voluntarily give my consent to be a participant in this research study.

Name and Signature of participant
Date:

Name and signature of investigator
Date:

Name and signature of witness (if required, as in case illiterate participants and consent by LAR)
Date:

**PROJECT PROPOSAL**

**Role of Adjuvant Yoga Therapy in Managing Withdrawal Symptoms of Patients with Opioid Use Disorder**

**Introduction**:

Opioid use disorder is characterized by chronic use of opioids such as heroin, morphine, codeine, fentanyl, and synthetic opioids like oxycodone which causes clinically significant impairment. It involves an intense desire to use opioids, increased opioid tolerance, and withdrawal syndrome when discontinued. Opioid use disorders affect over 16 million people worldwide and over 120,000 deaths worldwide annually occur due to opioids.(1)

Withdrawal from opioids is characterized by specific problematic behavioral change, with physiologic and cognitive components. The opioid withdrawal syndrome is a collection of clinical signs and symptoms such as hypertension, tachycardia, insomnia, mydriasis, piloerection, lacrimation, excessive sweating, rhinorrhoea, yawning, nausea, vomiting and diarrhea, anxiety, shivering, body aches, easily agitated, craving, hyperactivity, rapid breathing.(2,3) The time course of withdrawal is dependent on the half-life of the opioid used; it may last from 5 to 14 days.(2)

World health organisation recommended three medication treatment for withdrawal symptoms.(4) They are 1) opioid receptor full agonist methadone, 2) opioid receptor antagonist naltrexone 3) opioid receptor partial agonist buprenorphine (available as sublingual or buccal tablets or films, a skin patch). Patients treated with Buprenorphine experienced less severe withdrawal when compared with other drugs(5). Although buprenorphine is an ideal drug of choice, it alone does not control withdrawal symptoms, so ancillary medications such as clonazepam (sedative-hypnotics) for anxiety, trazodone and zolpidem (act via GABA) for sleep, ibuprofen (NSAID) for muscle cramps, bismuth subsalicylate for diarrhea, Ondansetron and prochlorperazine for nausea and vomiting are also used in the management of withdrawal symptoms.(6)

Sleep disturbance is common and often severe during opioid withdrawal. Sleep disturbance includes reduced total sleep time, continuity and quality which causes poor treatment outcomes.(7) Sleep and substance use are found to be interrelated, there is a lot of sleep disturbance evident in patients with opioid use disorder before and after treatment.(8) Sleep and substance use are found to be interrelated, there is a lot of sleep disturbance evident in patients with opioid use disorder before and after treatment. There is limited research in understanding HRV changes during the withdrawal phase which shows a significant decrease in cardiac vagal tone following naloxone administration in opioid-dependent participants.(9) Pain is one of the risk factors for increased severity of withdrawal symptoms.(10) Buprenorphine is found to be effective in managing pain-related symptoms well when compared with other drugs.(11) Opioid withdrawal is also associated with anxiety and other symptoms related to sympathetic outflow which are usually treated with drugs such as lofexidine and clonidine(12).

Opioid partial agonist buprenorphine peak of action is 30-60 min and has a duration of action up to 72 hrs. for high doses.(13) Increasing dose of buprenorphine may produce typical side effects such as constipation, insomnia, weight gain, hormonal changes, cardiac arrhythmias

etc.(5) Buprenorphine is a costly drug and not easily accessible, it is only available at designated Opioid Substitution Therapy (OST) centres.

Yoga as an adjuvant could be an effective treatment for early recovery from various withdrawal symptoms. Yoga involves a combination of physical postures (asanas), breathing exercises (pranayama), meditation and relaxation. Evidence suggests that yoga could be effective in treating various withdrawal symptoms such as sleep disturbance, anxiety, body aches, craving, agitation, etc. Yoga effectively improves total sleep duration and sleep quality.(14) Yoga affects mood and improves the quality of life in patients suffering from substance use disorder, which is usually low in them when compared with the normal population.(15) Yoga could show improvement in symptoms like pain and anxiety immediately after the yoga session.(16) Studies suggest a reduction in anxiety in practitioners of yoga doing yoga at least once a week for 6 weeks.(17) Yoga has an effect on the autonomic nervous system several studies suggest an increase in parasympathetic activity in individuals practicing yoga.(18) A validated yoga module has been developed and found to be feasible, safe and potentially useful in reducing withdrawal symptoms and cravings of opioid use disorder patients.(19)

In the current study, we hypothesize that Yoga as an add-on therapy will show a significant form of recovery from withdrawal symptoms of opioid use disorder when compared to treatment only group.

**Aim:**

To study the impact of Yoga as an add-on therapy in recovery from withdrawal symptoms and autonomic regulation of opioid use disorder when compared to standard treatment alone.

**Objectives:**

- Primary objective:
    1. To evaluate the effect of Yoga on time to recovery from opioid withdrawal symptoms in patients with OUD using the and Clinical Opiate Withdrawal Scale (COWS) and heart rate variability parameters reflecting autonomic regulation.

- Secondary objective**:**
  To access the changes following clinical measures in yoga as an add-on therapy group when compared with the standard treatment-alone group.

    1. Anxiety (HAM-A)
    2. Sleep latency
    3. Brief Pain Inventory

**Hypothesis:**

- Null Hypothesis:
  Yoga as an add-on therapy won't show a significant form of recovery from withdrawal symptoms of opioid use disorder and improved autonomic regulation when compared to treatment only group.

- Alternate Hypothesis:
  Yoga as an add-on therapy will show a significant form of recovery from withdrawal symptoms of opioid use disorder and improved autonomic regulation when compared to treatment only group.

**Methodology:**

- Age: 18 to 50 years.
- Sex: Both male and female.
- Sample size: 60 (30 patients in Yoga + Treatment as usual group and 30 patients in treatment only group).
- Source of Subject: Inpatient services run by the CAM & Department of Integrative Medicine, National Institute of Mental Health and Neurosciences (NIMHANS), Bengaluru, India

**Inclusion criteria:**

- Diagnosis of Opioid use disorder.
- Subjects with mild to moderate severity of withdrawal symptoms (Clinical Opiate Withdrawal Scale: COWS scores between 4 - 24).
- Subjects who got admitted into CAM, NIMHANS and started treatment with Buprenorphine.
- Age range: 18 to 50 years.
- Both genders.
- Written informed consent.

**Exclusion criteria:**

- COWS score more than 25.
- Patients not willing to give informed consent.
- History of current medical illness that may significantly affect brain functions
- Patients with intractable pain due to other diseases and those on prescribed opioids for the pain of other medical conditions
- Impaired general intellectual functioning (Mini-Mental Status Exam, MMSE below 24).
- Past history of head injury or concomitant severe medical conditions.
- With co-morbid (alcohol/benzodiazepine/cannabis or multiple) substance dependence except nicotine.
- Prior exposure to specific yoga or other mind-body practices for OUD within past 6 months.
- Pregnant or post-partum subjects.

**Assessments:**

- Clinical Opiate Withdrawal Scale (COWS)

- Heart Rate Variability (HRV)
- Hamilton anxiety rating scale (HAM-A)
- Brief Pain Inventory (BPI)
- Sleep Latency
- Yoga performance assessment (YPA)

**Study design:**

Patients diagnosed with opioid use disorder are assessed for severity of withdrawal symptoms using Clinical Opiate Withdrawal Scale (COWS). The patients with mild to moderate severity of withdrawal symptoms and taking buprenorphine for management of symptoms are randomized into two groups that is Yoga + standard treatment group and Treatment only group. Duration of intervention is 2 weeks. Clinical history will be done at baseline. Assessments will be done at the baseline and at the end of the intervention, for those who are willing to participate in the study. Safety assessments and adverse events will be monitored and appropriately documented for the duration of study period. <u>The medications prescribed and taken by the patients other than buprenorphine will be documented in both the groups during the study period.</u>

**Schematic representation:**

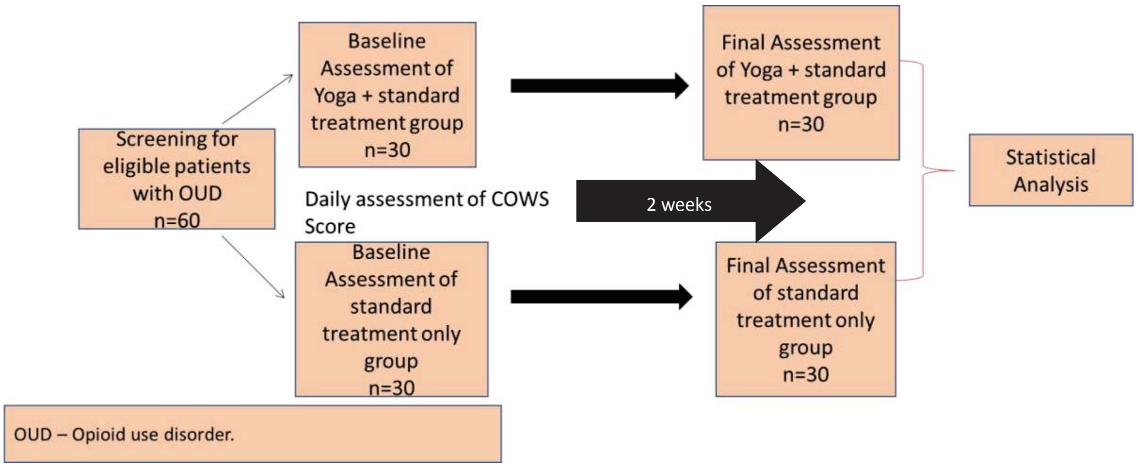

**Treatment:**

Validated Yoga Module for 2 weeks (10 supervised sessions over 2 weeks- 45minutes/session) (Developed by Hemant Bhargav et al., 2021 at NIMHANS)

The module to be taught and practiced under the supervision of a trained Yoga therapist only.

Yoga module for substance use disorder during Acute withdrawal phase.

| S.no | Practice (Sanskrit) | Practice (English) | Rounds | Time (minutes) |
|---|---|---|---|---|
| RELAXATION TECHNIQUE | | | | |
| 1. | Takshan shithalikaran upaya | Instant Relaxation Technique | 2 | 4 |
| BREATHING PRACTICES | | | | |
| 1. | Pawanmuktasana kriya | Wind releasing pose breathing | 5 | 5 |
| 2. | Udarashvasana kriya | Deep abdominal breathing | 5 | 2 |
| 3. | Makarasana shvasana kriya | Crocodile pose breathing | 5 | 2 |
| 4. | Bhujangasana shvasana kriya | Cobra pose breathing | 5 | 2 |
| 5. | Naukasana shvasana kriya | Boat pose breathing | 5 | 2 |
| 6. | Marjariasna kriya | Tiger stretch breathing | 5 | 2 |
| SPECIFIC ASANAS | | | | |
| 1. | Uttana padasana | Straight leg raising | 5 | 2 |
| 2. | Titali asana | Butterfly | 1 | 2 |
| 3. | Trikonasana | Side bending | 5 | 2 |
| SECTIONAL BREATHING | | | | |
| 1. | Vibhagiya pranayama | Chin mudra | 5 | 2 |
| 2. | Vibhagiya pranayama | Chinmaya mudra | 5 | 2 |
| 3. | Vibhagiya pranayama | Adi mudra | 5 | 2 |
| PRANAYAMA | | | | |
| 1. | Nadishuddhi | Alternate nostril breathing | 9 | 3 |
| 2. | Bhastrika | Bellows breath | 2 | 2 |
| 3. | Bhramari | Humming bee breath | 9 | 2 |
| RELAXATION | | | | |
| 1. | Sampurna vishranti paddati in shavasana with AUM chants | Deep relaxation technique | 1 | 5 |

**Statistical analysis:**

- Data would be analyzed using R statistical software (Version 4.4.1). Based on the distribution and variance appropriate statistical tests will be used for analysis.
- Baseline continuous data will be analyzed using an independent sample t-test. Baseline categorical data will be analyzed using the chi-square test. Data will be checked for normality, for longitudinal analysis, a linear mixed model will be used.
- If the data is not normally distributed and the samples are small, a nonparametric test is appropriate, and to compare two groups Mann Whitney U test is performed.

- Primary longitudinal outcomes will be analyzed using Linear Mixed-Effects Models (LMM) with the lme4 package.
- Time to recovery from withdrawal symptoms will be analyzed using survival analysis.

**Ethical considerations:**

- Subjects will be informed about all the required procedures of the study and written informed consent will be obtained.
- The investigator will withdraw the subject from participating in the research if; the subject does not fall under the inclusion criteria or if the subject becomes ill during the study.
- The study will be registered under CTRI.
- No financial obligations for the subject. No charges will be collected for the yoga therapy and the assessments.
- Confidentiality of all information will be assured and maintained.
- Subjects will have the right to withdraw consent at any stage.
- Approval from the ethical committee of NIMHANS will be sought.

**Financial obligations:**

- No financial obligations for the subject. All the treatment procedures, assessments, and investigations will be done at free of cost.
- Confidentiality of all information will be assured and maintained.
- Subjects will have the right to withdraw consent at any stage.
- Approval from the Ethical committee of NIMHANS will be sought.

**Implications of the study:**

- Yoga therapy can integrate with standard treatment in managing Opioid withdrawal symptoms for the early recovery of patients.
- Yoga therapy could not only help in managing withdrawal symptoms but also can act as a lifestyle intervention that is more culturally adaptable, accessible, and cost-effective.

**References:**

1. Dydyk AM, Jain NK, Gupta M. Opioid Use Disorder. In: StatPearls [Internet]. Treasure Island (FL): StatPearls Publishing; 2022 [cited 2023 Jan 18]. Available from: http://www.ncbi.nlm.nih.gov/books/NBK553166/

2. Kosten TR, Baxter LE. Review article: Effective management of opioid withdrawal symptoms: A gateway to opioid dependence treatment. Am J Addict. 2019 Feb;28(2):55–62.

3. Wesson DR, Ling W. The Clinical Opiate Withdrawal Scale (COWS). J Psychoactive Drugs. 2003;35(2):253–9.

4. Abuse WHOD of MH and S, Organization WH. Guidelines for the Psychosocially Assisted Pharmacological Treatment of Opioid Dependence. World Health Organization; 2009. 133 p.

5. Gowing L, Ali R, White JM, Mbewe D. Buprenorphine for managing opioid withdrawal. Cochrane Database Syst Rev. 2017 Feb 21;2017(2):CD002025.

6. Pitha PM, Carter WA. The DEAE dextran:polyriboinosinate-polyribocytidylate complex: physical properties and interferon induction. Virology. 1971 Sep 1;45(3):777–81.

7. Huhn AS, Finan PH. Sleep disturbance as a therapeutic target to improve opioid use disorder treatment. Exp Clin Psychopharmacol. 2022 Dec;30(6):1024–35.

8. Fathi HR, Yoonessi A, Khatibi A, Rezaeitalab F, Rezaei-Ardani A. Crosstalk between Sleep Disturbance and Opioid Use Disorder: A Narrative Review. Addict Health. 2020 Apr;12(2):140–58.

9. Levin CJ, Wai JM, Jones JD, Comer SD. Changes in cardiac vagal tone as measured by heart rate variability during naloxone-induced opioid withdrawal. Drug Alcohol Depend. 2019 Nov 1;204:107538.

10. Ware OD, Ellis JD, Dunn KE, Hobelmann JG, Finan P, Huhn AS. The association of chronic pain and opioid withdrawal in men and women with opioid use disorder. Drug Alcohol Depend. 2022 Nov 1;240:109631.

11. Srivastava AB, Mariani JJ, Levin FR. New directions in the treatment of opioid withdrawal. Lancet Lond Engl. 2020 Jun 20;395(10241):1938–48.

12. Urits I, Patel A, Zusman R, Virgen CG, Mousa M, Berger AA, et al. A Comprehensive Update of Lofexidine for the Management of Opioid Withdrawal Symptoms. Psychopharmacol Bull. 2020 Jul 23;50(3):76–96.

13. Cisewski DH, Santos C, Koyfman A, Long B. Approach to buprenorphine use for opioid withdrawal treatment in the emergency setting. Am J Emerg Med. 2019 Jan;37(1):143–50.

14. Wang WL, Chen KH, Pan YC, Yang SN, Chan YY. The effect of yoga on sleep quality and insomnia in women with sleep problems: a systematic review and meta-analysis. BMC Psychiatry. 2020 May 1;20(1):195.

15. Dhawan A, Chopra A, Jain R, Yadav D, Vedamurthachar null. Effectiveness of yogic breathing intervention on quality of life of opioid dependent users. Int J Yoga. 2015;8(2):144–7.

16. Uebelacker LA, Van Noppen D, Tremont G, Bailey G, Abrantes A, Stein M. A pilot study assessing acceptability and feasibility of hatha yoga for chronic pain in people receiving opioid agonist therapy for opioid use disorder. J Subst Abuse Treat. 2019 Oct;105:19–27.

17. Lemay V, Hoolahan J, Buchanan A. Impact of a Yoga and Meditation Intervention on Students' Stress and Anxiety Levels. Am J Pharm Educ. 2019 Jun;83(5):7001.

18. Streeter CC, Gerbarg PL, Saper RB, Ciraulo DA, Brown RP. Effects of yoga on the autonomic nervous system, gamma-aminobutyric-acid, and allostasis in epilepsy, depression, and post-traumatic stress disorder. Med Hypotheses. 2012 May;78(5):571–9.

19. Development, Validation, and Feasibility Testing of a Yoga Module for Opioid Use Disorder - PubMed [Internet]. [cited 2023 Jan 18]. Available from: https://pubmed.ncbi.nlm.nih.gov/34237026/
