# Supplementary material for: Yoga for Opioid Withdrawal and Autonomic Regulation: A Randomized Clinical Trial
Source: JAMA Psychiatry. 2026 Jan 7;83(3):238–46. doi: 10.1001/jamapsychiatry.2025.3863 (PMC12780978; doi:10.1001/jamapsychiatry.2025.3863)
Supplement: Supplement 2. — eMethods 1. Detailed HRV Preprocessing Protocol eMethods 2. Standard Buprenorphine Protocol eMethods 3. Mediation Analysis Script eTable 1. Key Physiological Parameters by Group and Time eTable 2. Yoga Module for Opioid Withdrawal - Practice Details and Rationale eFigure 1. Linear Mixed-Effects Model Plots Showing Changes in Clinical Outcome Measures Between Yoga and Control Groups eFigure 2. Yoga Performance Assessment (YPA) Scores at Day 7 and Day 15 eFigure 3. Linear Mixed-Effects Model Plots Showing Changes in Heart Rate Variability (HRV) During the Pranayama Protocol Between Yoga and Control Groups [file jamapsychiatry-e253863-s002.pdf]

Supplementary Online Content

Goutham S, Bhargav H, Holla B, et al. Yoga for opioid withdrawal and autonomic regulation: a randomized clinical trial. *JAMA Psychiatry*. Published online January 7, 2026. doi:10.1001/jamapsychiatry.2025.3863

eMethods 1. Detailed HRV Preprocessing Protocol
eMethods 2. Standard Buprenorphine Protocol
eMethods 3. Mediation Analysis Script
eTable 1. Key Physiological Parameters by Group and Time
eTable 2. Yoga Module for Opioid Withdrawal - Practice Details and Rationale
eFigure 1. Linear Mixed-Effects Model Plots Showing Changes in Clinical Outcome Measures Between Yoga and Control Groups
eFigure 2. Yoga Performance Assessment (YPA) Scores at Day 7 and Day 15
eFigure 3. Linear Mixed-Effects Model Plots Showing Changes in Heart Rate Variability (HRV) During the Pranayama Protocol Between Yoga and Control Groups

This supplemental material has been provided by the authors to give readers additional information about their work.

© 2026 Goutham S et al. JAMA Psychiatry.

eMethods 1. Detailed HRV Preprocessing Protocol

ECG Preprocessing and HRV Analysis

ECG preprocessing was performed using **NeuroKit2** (Makowski et al., 2021). Raw ECG signals were first cleaned with the ecg_clean function (default *neurokit* method: 0.5 Hz high-pass Butterworth filter with powerline suppression).

R-peak detection and artifact correction. R-peaks were identified with ecg_peaks using automated artifact correction (correct_artifacts=True), which implements the Kubios-style algorithm of Lipponen and Tarvainen (2019). Beats were classified as ectopic, missed, extra, or long/short based on deviations in RR intervals and local medians. Corrections were applied locally: extra beats were removed, missed beats were interpolated by inserting an R-peak at the expected midpoint, and implausible or ectopic intervals were replaced with interpolated values from surrounding beats. This yielded a corrected **normal-to-normal (NN) interval series**, preserving continuity rather than discarding segments.

Refinement and quality control. Peaks were further refined using signal_fixpeaks in iterative mode with robust thresholds of ±3.5 SD of RR intervals. Signal quality was indexed with ecg_quality ("averageQRS" method), which quantifies each beat's similarity to the average QRS template (1 = most similar, 0 = least). As an additional QC measure, we calculated the percentage of retained beats after artifact correction, with a conservative exclusion threshold of <98% retained beats or poor averageQRS values. In this dataset, all recordings exceeded these thresholds, and no epochs were excluded.

HRV feature extraction. From the corrected NN series, time-domain indices were derived with hrv_time. Frequency-domain metrics were computed using hrv_frequency with multitaper spectral estimation (Thomson, 1982) without normalization. LF (0.04–0.15 Hz) and HF (0.15–0.40 Hz) bands were defined per Task Force recommendations (1996). Relative indices LF% and HF% were computed as LF and HF power expressed as a percentage of total power minus VLF. The LF/HF ratio was log-transformed to address skewness; estimates were obtained on the log scale, and predictions were back-transformed to the original ratio scale for interpretation.

eMethods 2. Standard Buprenorphine Protocol

All participants received buprenorphine following institutional protocols aligned with the National AIDS Control Organisation (NACO) Clinical Practice Guidelines (Rao et al., 2014). For heroin users, induction and titration followed NACO guidance. For tapentadol users, the protocol was similar but required lower dosing, as described in a recent publication from our centre (Shivaprakash et al., 2025; PubMed ID: 40181877).

Induction criteria: Clinical Opiate Withdrawal Scale (COWS) score ≥6.
Starting dose: 0.4 mg sublingual, titrated every 4–6 hours based on withdrawal severity.

© 2026 Goutham S et al. JAMA Psychiatry.

Median first-day dose: 0.8 mg (IQR = 0.4–2 mg).
Median maintenance dose: 4 mg (IQR = 2–8 mg).
Target dose: 2–8 mg/day, individualized based on craving and withdrawal severity.
Maximum dose: 8 mg/day (reserved for severe cases).
Physician training: All prescribers had completed standardized training in opioid substitution therapy as per NACO certification.
Monitoring: Daily COWS assessments guided dose adjustments.
Protocol adherence: No significant deviations from protocol occurred during the study.

References:

1. Lipponen JA, Tarvainen MP. A robust algorithm for heart rate variability time series artefact correction using novel beat classification. J Med Eng Technol. 2019 Apr;43(3):173-181. doi: 10.1080/03091902.2019.1640306. Epub 2019 Jul 17. PMID: 31314618.
2. Rao R, Agrawal A, Ambekar A. Opioid substitution therapy under National AIDS Control Programme: Clinical practice guidelines for treatment with buprenorphine. Department of AIDS Control, Ministry of Health and Family Welfare, Government of India, New Delhi. 2014.
3. Shivaprakash P, Shukla L, Joshi S, Mahadevan J, Kandasamy A, Chand PK, Benegal V, Murthy P. Tapentadol as a drug of abuse - A preliminary report. Indian J Psychiatry. 2025 Feb;67(2):256-259. doi: 10.4103/indianjpsychiatry.indianjpsychiatry_794_24. Epub 2025 Feb 19. PMID: 40181877; PMCID: PMC11964174.

eMethods 3. Mediation Analysis Script

```
# Requires: survival, tibble, dplyr
cox_mediation_boot <- function(
  dat, mediators,
  id = "id", treat = "treat", time = "time", status = "status",
  ref = NULL, B = 2000, seed = 123, ties = "efron"
) {
  set.seed(seed)
  dat[[treat]] <- if (!is.factor(dat[[treat]])) factor(dat[[treat]]) else dat[[treat]]
  if (!is.null(ref)) dat[[treat]] <- stats::relevel(dat[[treat]], ref = ref)

  qci <- function(x) {
    x <- x[is.finite(x)]
    if (length(x) < 20) return(c(NA, NA))
    stats::quantile(x, c(.025, .975))
  }
```

© 2026 Goutham S et al. JAMA Psychiatry.

```
  run_one <- function(med) {
    d <- dat[stats::complete.cases(dat[, c(id, treat, time, status, med)]), ]
    ids <- unique(d[[id]]); n_ids <- length(ids)

    a <- stats::coef(stats::lm(stats::as.formula(paste(med, "~", treat)), data = d))[treat]
    fit_tot <- survival::coxph(survival::Surv(d[[time]], d[[status]]) ~ d[[treat]], ties = ties)
    fit_dir <- survival::coxph(survival::Surv(d[[time]], d[[status]]) ~ d[[treat]] + d[[med]], ties
= ties)

    b       <- unname(stats::coef(fit_dir)["d[[med]]"])
    tot_log  <- unname(stats::coef(fit_tot)["d[[treat]]"])
    dir_log  <- unname(stats::coef(fit_dir)["d[[treat]]"])
    ind_log  <- a * b
    prop_med <- if (abs(tot_log) < 1e-8) NA_real_ else (tot_log - dir_log) / tot_log

    boot_ind <- boot_tot <- boot_dir <- boot_prp <- numeric(B)
    for (bti in seq_len(B)) {
      samp <- sample(ids, n_ids, replace = TRUE)
      db <- d[d[[id]] %in% samp, , drop = FALSE]

      m_b <- try(stats::lm(stats::as.formula(paste(med, "~", treat)), data = db), TRUE)
      t_b <- try(survival::coxph(survival::Surv(db[[time]], db[[status]]) ~ db[[treat]], ties =
ties), TRUE)
      d_b <- try(survival::coxph(survival::Surv(db[[time]], db[[status]]) ~ db[[treat]] +
db[[med]], ties = ties), TRUE)

      ok <- !(inherits(m_b,"try-error") || inherits(t_b,"try-error") || inherits(d_b,"try-error")) &&
            all(c("db[[treat]]","db[[med]]") %in% names(stats::coef(d_b))) &&
            "db[[treat]]" %in% names(stats::coef(t_b))
      if (!ok) next
      a_b <- stats::coef(m_b)[treat]
      b_b <- unname(stats::coef(d_b)["db[[med]]"])
      tot <- unname(stats::coef(t_b)["db[[treat]]"])
      dir <- unname(stats::coef(d_b)["db[[treat]]"])
      boot_ind[bti] <- a_b * b_b
      boot_tot[bti] <- tot
      boot_dir[bti] <- dir
      boot_prp[bti] <- if (abs(tot) < 1e-8) NA_real_ else (tot - dir) / tot
    }

    ci_ind <- qci(boot_ind); ci_tot <- qci(boot_tot)
    ci_dir <- qci(boot_dir); ci_prp <- qci(boot_prp)

    tibble::tibble(
      mediator = med, n_ids = n_ids, B = B,
      indirect_logHR = ind_log, indirect_logHR_lo = ci_ind[1], indirect_logHR_hi = ci_ind[2],
      indirect_HR = exp(ind_log), indirect_HR_lo = exp(ci_ind[1]), indirect_HR_hi =
exp(ci_ind[2]),
      total_logHR = tot_log, total_logHR_lo = ci_tot[1], total_logHR_hi = ci_tot[2],
```

© 2026 Goutham S et al. JAMA Psychiatry.

```
    total_HR = exp(tot_log), total_HR_lo = exp(ci_tot[1]), total_HR_hi = exp(ci_tot[2]),
    direct_logHR = dir_log, direct_logHR_lo = ci_dir[1], direct_logHR_hi = ci_dir[2],
    direct_HR = exp(dir_log), direct_HR_lo = exp(ci_dir[1]), direct_HR_hi = exp(ci_dir[2]),
    prop_med = prop_med, prop_med_lo = ci_prp[1], prop_med_hi = ci_prp[2]
   )
  }

  dplyr::bind_rows(lapply(mediators, run_one))
}

# dat columns: id, treat, time, status, dHF, dLF, dLFHF
res <- cox_mediation_boot(
  dat, mediators = c("dHF","dLF","dLFHF"),
  id = "id", treat = "treat", time = "time", status = "status",
  ref = "TAU", B = 500, seed = 99
)
print(res)
```

© 2026 Goutham S et al. JAMA Psychiatry.

eTable 1. Key Physiological Parameters by Group and Time

	**TAU (D01) (N=29)**	**YAT (D01) (N=30)**	**TAU (D15) (N=26)**	**YAT (D15) (N=29)**
ECG Quality	0.91 (0.06)	0.91 (0.05)	0.91 (0.07)	0.90 (0.06)
Percentage of Beats Retained for Analysis	99.42 (0.07)	99.44 (0.07)	99.40 (0.12)	99.44 (0.09)
Mean Heart Rate (bpm)	89.42 (10.57)	86.14 (11.43)	92.84 (18.39)	86.84 (13.73)
Mean Respiration Rate (breaths/min)	20.17 (6.07)	21.30 (6.97)	19.75 (5.22)	20.62 (7.23)
Mean NN Interval (ms)	683.13 (87.98)	714.16 (124.88)	678.89 (162.43)	710.22 (116.93)
SDNN (ms)	39.54 (14.51)	38.07 (18.03)	43.52 (25.99)	33.68 (18.40)
RMSSD (ms)	17.92 (8.96)	18.42 (12.39)	22.25 (19.30)	19.19 (14.20)
TINN (ms)	158.67 (56.61)	161.46 (76.20)	171.57 (106.19)	143.59 (91.31)
Low Frequency Power (%)	77.60 (10.07)	81.27 (9.94)	77.75 (10.79)	63.61 (18.90)
High Frequency Power (%)	20.64 (10.08)	16.96 (9.44)	20.71 (10.46)	33.17 (17.51)
LF/HF Ratio	5.50 (4.77)	6.64 (3.85)	5.87 (5.76)	3.66 (5.50)
SD1 (ms)	12.68 (6.34)	13.03 (8.77)	15.75 (13.66)	13.58 (10.05)

© 2026 Goutham S et al. JAMA Psychiatry.

SD2 (ms)	54.35 (19.73)	52.09 (24.23)	59.35 (34.33)	45.36 (24.50)
SD1/SD2 Ratio	0.23 (0.06)	0.24 (0.07)	0.24 (0.06)	0.30 (0.11)

Data are presented as Mean (SD). ECG Quality (0-1) is derived from the average QRS method, where scores near 1 indicate good quality. Abbreviations: ECG, electrocardiogram; HF, high frequency; HR, heart rate; HRV, heart rate variability; LF, low frequency; LF/HF, low frequency to high frequency ratio; NN, normal-to-normal interval; RMSSD, root mean square of successive differences; RSP, respiration; SD1, standard deviation of short-term interval variability; SD2, standard deviation of long-term interval variability; SDNN, standard deviation of normal-to-normal intervals; TINN, triangular interpolation of NN interval histogram.

© 2026 Goutham S et al. JAMA Psychiatry.

eTable 2. Yoga Module for Opioid Withdrawal - Practice Details and Rationale

Specific Practice	**Position**	**Duration**	**Rounds/ Cycles**	**Rationale for Use**
Opening				
Starting prayer	Sitting	1 min	1	Establishes mindful intention; reduces anxiety through ritual behavior
Physical Postures				
Instant relaxation technique	Supine	4 min	2	Activates parasympathetic nervous system; counters sympathetic hyperactivity in withdrawal
Straight leg raising (alternate)	Supine	3 min	5 each leg	Improves circulation; strengthens core; aids venous return compromised by prolonged inactivity
Pavanamuktasana (alternate)	Supine	2 min	5 each leg	Stimulates digestive organs; relieves constipation common in opioid withdrawal
Makarasana (alternate)	Prone	3 min	5 each leg	Promotes natural diaphragmatic breathing; relieves lower back tension from muscle aches
Breathing Practices				
Deep abdominal breathing	Supine	2 min	5 cycles	Enhances vagal tone; reduces respiratory distress; 1:2 ratio maximizes parasympathetic activation
Sectional breathing (Vibhagiya) - Chin, Chinmaya, Adi Mudras	Sitting	6 min	5 cycles in each mudra	Systematic respiratory training targeting all lung segments; 4:16:8 ratio builds breath retention and autonomic balance across diaphragmatic, thoracic, and clavicular regions
Bhastrika (slow)	Sitting	3 min	2 sets of 10	Generates internal heat; counters temperature dysregulation in withdrawal
Nadi Shuddhi	Sitting	3 min	9 cycles	Balances sympathetic/parasympathetic activity; traditional anxiety reducer

© 2026 Goutham S et al. JAMA Psychiatry.

Bhramari	Sitting/ Supine	3 min	9 cycles	Vibrations stimulate vagus nerve; sound therapy reduces rumination and craving
Relaxation				
Guided yogic relaxation	Supine	9 min	1 session	Systematic muscle relaxation; positive affirmations counter negative thought patterns
Closing				
End prayer	Sitting	1 min	1	Provides closure and sense of completion; reinforces positive intention

© 2026 Goutham S et al. JAMA Psychiatry.

eFigure 1. Linear Mixed-Effects Model Plots showing changes in clinical outcome measures Changes Between Yoga and Control Groups.

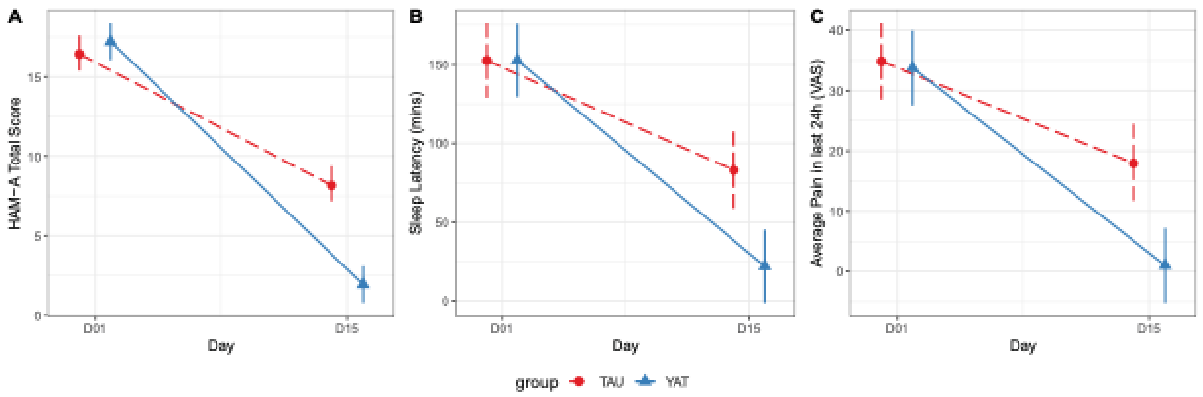

eFigure 1 legend: Yoga Add-on Therapy (YAT; blue line); Treatment-as-Usual (TAU; red dashed line) groups. ***Panels (A-C)*** *show changes from baseline (Day 1) to post-intervention (Day 15) in (A) HAM-A; Hamilton Anxiety Rating Scale; (B) Sleep Latency and (C) Average pain in last 24 VAS; Visual Analogue Scale (BPI average pain severity (0-10 scale), converted to 0-100 VAS for analysis consistency.). A significant group-by-time interaction effect was observed for all three measures.*

© 2026 Goutham S et al. JAMA Psychiatry.

eFigure 2. Yoga Performance Assessment (YPA) Scores at Day 7 and Day 15

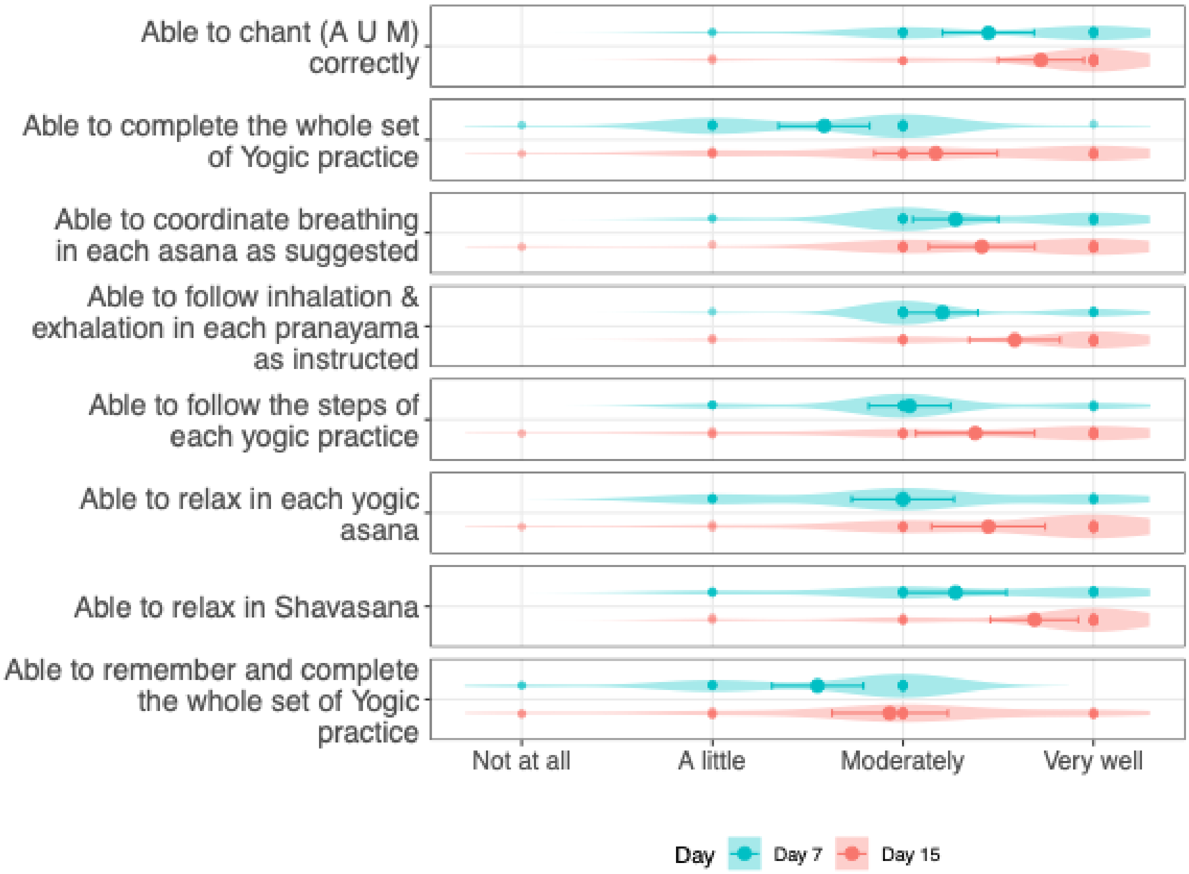

eFigure 2 legend: Distribution of participant responses to the Yoga Performance Assessment (YPA) questionnaire at the end of week 1 (Panel A: Day 7) and week 2 (Panel B: Day 15).

Each horizontal bar corresponds to a specific item on the YPA and shows the percentage of participants who selected each response category ("Not at all," "A little," "Moderately," or "Very well"). The clear shift in responses towards "Moderately" and "Very well" from Day 7 to Day 15 indicates that participants' proficiency and confidence in performing the yoga module significantly improved over the two-week intervention period.

© 2026 Goutham S et al. JAMA Psychiatry.

eFigure 3 (A to C). Linear Mixed-Effects Model Plots showing changes in Heart Rate Variability (HRV) Changes Between Yoga and Control Groups during the pranayama protocol.

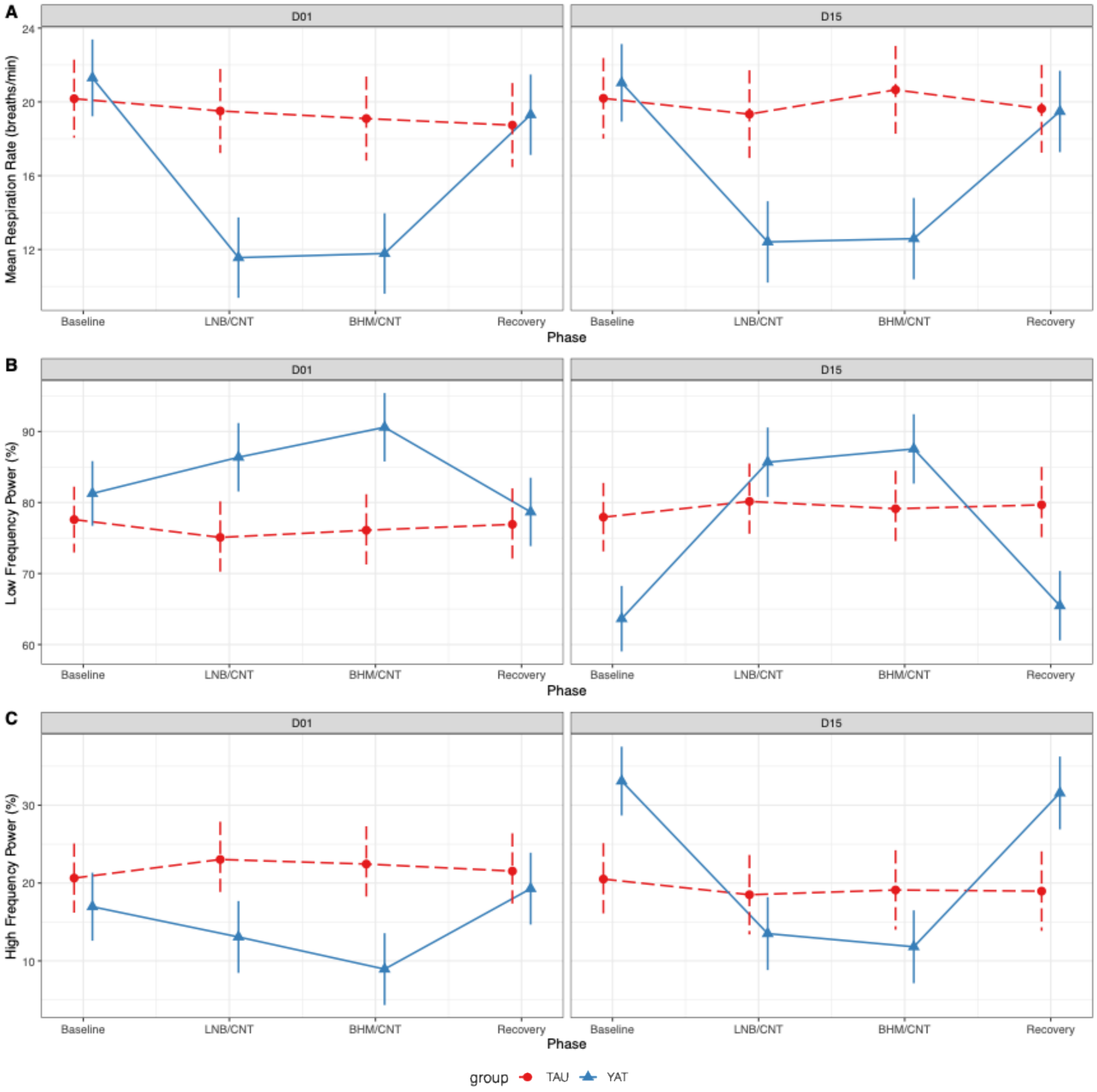

eFigure 3 legend: Estimated marginal means with 95% confidence intervals showing Yoga Add-on Therapy (YAT; blue line); Treatment-as-Usual (TAU; red dashed line) groups during the immediate effect of the pranayama protocol on Day 1 (left) and Day 15 (right) for Mean Respiration Rate (breaths/min) (A), normalized Low Frequency (LF) power (B), and normalized High Frequency (HF) power (C). Phases include Baseline Rest, Left Nostril Breathing (LNB)/Control (CNT), Bhramari pranayama (BHM)/CNT, and Recovery. Note: i) Group × time effects were tested on resting baseline HRV, ii) LNB, BHM, and recovery were analyzed descriptively as process measures of acute pranayama effects, not as part of the primary statistical model.

© 2026 Goutham S et al. JAMA Psychiatry.
