# Supplementary material for: Yoga for Opioid Withdrawal and Autonomic Regulation: A Randomized Clinical Trial
Source: JAMA Psychiatry. 2026 Jan 7;83(3):238–46. doi: 10.1001/jamapsychiatry.2025.3863 (PMC12780978; doi:10.1001/jamapsychiatry.2025.3863)
Supplement: Supplement 3. — Data Sharing Statement [file jamapsychiatry-e253863-s003.pdf]

Data Sharing Statement

Goutham. Yoga for Opioid Withdrawal and Autonomic Regulation. *JAMA Psychiatry*. Published January 07, 2026. doi:10.1001/jamapsychiatry.2025.3863

Data
Additional Information: CTRI/2023/04/051302
Data available: Yes
Data types: Deidentified participant data
How to access data: drbhargav.nimhans@gmail.com
When available: With publication

Supporting Documents
Document types: None

Additional Information
Who can access the data: researchers whose proposed use of the data has been approved
Types of analyses: for any purpose
Mechanisms of data availability: after approval of a proposal
Any additional restrictions: None
